# Robust Acquisition of Spatial Transcriptional Programs in Tissues With Immunofluorescence-Guided Laser Capture Microdissection

**DOI:** 10.3389/fcell.2022.853188

**Published:** 2022-03-25

**Authors:** Xiaodan Zhang, Chuansheng Hu, Chen Huang, Ying Wei, Xiaowei Li, Miaomiao Hu, Hua Li, Ji Wu, Daniel M. Czajkowsky, Yan Guo, Zhifeng Shao

**Affiliations:** ^1^ State Key Laboratory of Oncogenes and Related Genes, School of Biomedical Engineering, Shanghai Jiao Tong University, Shanghai, China; ^2^ Department of Gastrointestinal Surgery, Renji Hospital, School of Medicine, Shanghai Jiao Tong University, Shanghai, China; ^3^ Bio-X Institute, Shanghai Jiao Tong University, Shanghai, China

**Keywords:** spatial transcriptome, laser capture microdissection (LCM), immunofluorescence, RNA, lacteal, RNAlater

## Abstract

The functioning of tissues is fundamentally dependent upon not only the phenotypes of the constituent cells but also their spatial organization in the tissue, as local interactions precipitate intra-cellular events that often lead to changes in expression. However, our understanding of these processes in tissues, whether healthy or diseased, is limited at present owing to the difficulty in acquiring comprehensive transcriptional programs of spatially- and phenotypically-defined cells *in situ*. Here we present a robust method based on immunofluorescence-guided laser capture microdissection (immuno-LCM-RNAseq) to acquire finely resolved transcriptional programs with as few as tens of cells from snap-frozen or RNAlater-treated clinical tissues sufficient to resolve even isoforms. The protocol is optimized to protect the RNA with a small molecule inhibitor, the ribonucleoside vanadyl complex (RVC), which thereby enables the typical time-consuming immunostaining and laser capture steps of this procedure during which RNA is usually severely degraded in existing approaches. The efficacy of this approach is exemplified by the characterization of differentially expressed genes between the mouse small intestine lacteal cells at the tip versus the main capillary body, including those that function in sensing and responding to local environmental cues to stimulate intra-cellular signalling. With the extensive repertoire of specific antibodies that are presently available, our method provides an unprecedented capability for the analysis of transcriptional networks and signalling pathways during development, pathogenesis, and aging of specific cell types within native tissues.

## Introduction

It is now well-recognized that the functioning of any tissue, whether healthy or diseased, is uniquely determined by the diverse constituent cells interacting within a highly structured three-dimensional architecture. Communications between neighbouring cells, interactions with the extracellular milieu, and proximity to vasculature are all well known to initiate intra-cellular events that can lead to significant changes in expression, fundamentally altering cell states and phenotypes in a spatially dependent manner. From the intricate configuration of cells within and between the functional domains of the brain, to the mosaic distribution of malignant and non-malignant cells within the tumor microenvironment, to the dynamic patterns of progenitor cells during embryogenesis, there is now wide appreciation of the significant influence of the local environment on cellular transcriptional profiles (Peng et al., 2019; Di Paolo et al., 2021; Marx 2021). Thus, techniques that can characterize the transcription programs of defined cells within a tissue whilst retaining their spatial information are of immense interest to better understand these events and their functional consequences (Crosetto et al., 2005; Moor and Itzkovitz 2017). Recent developments to this end can be generally divided into two broad categories: methods based on spatially resolved imaging of fluorescence *in situ* hybridization (FISH) and those based on spatial profiling of transcripts using next generation sequencing. For the former, while directly imaging the FISH signals provides a straightforward means of detection and quantification, the transcriptomes determined by these methods only include those transcripts that are fully annotated and for which suitable probes can be designed, which limits the detectable transcripts, particularly those from novel genes (Asp et al., 2020; Liao et al., 2021). This issue can be avoided with sequencing-based approaches, such as Slide-seq (Rodriques et al., 2019) and HDST (Vickovic et al., 2019), which employ tagged beads, and ExSeq (Alon et al., 2021), where sequencing is performed *in situ*. However, both bead-bound approaches and ExSeq can be significantly limited in their capture efficiency of transcripts, resulting in detection of only tens to hundreds of genes per cell (Rodriques et al., 2019; Vickovic et al., 2019; Alon et al., 2021). Furthermore, identifying individual cells in the tissue that are associated with bead-bound transcripts is less straightforward and often requires knowledge of the transcriptomes of the constituent cells in the tissue a priori, ideally acquired under the same (physiological) conditions. Moreover, owing to the enrichment at the 3’ end of the transcripts, the bead-based methods do not resolve transcript isoforms, which, it should be noted, is also a problem with the imaging-based approaches as well.

In this regard, the more traditional laser capture microdissection (LCM) (Emmert-Buck et al., 1996) combined with RNAseq is a powerful approach with its own unique strengths for the acquisition of spatial transcriptomes, since cells are directly selected from the tissue with complete knowledge of their spatial location for an unbiased delineation of their transcriptional program. Although identifying cells for LCM based on cell morphology alone is possible in some cases (Hawrylycz et al., 2012; Peng et al., 2016; Murray 2018), the vast majority of cell types in a tissue cannot be distinguished by morphology alone. Therefore, use of specific phenotype markers, especially immunofluorescence (IF)-based cell type identification, remains the most attractive strategy to acquire comprehensive transcriptional programs *in situ* with LCM (Fend et al., 1999; Murakami et al., 2000). To date, however, despite some commonly-held perceptions, there are, in fact, only limited successes of highly effective integration of IF with LCM for transcriptome acquisition (Tangrea et al., 2011a; Murray 2018; Liao et al., 2021; Rao et al., 2021). This difficulty is primarily owing to the fact that RNA is often significantly degraded during IF labelling as a result of the activities of both intrinsic and ubiquitous exogenous RNases, even with the use of potent recombinant RNase inhibitors (Fend et al., 1999; Murakami et al., 2000; Tangrea et al., 2011b). Several strategies to overcome this problem have been proposed, including drastically shortening the incubation time during labelling while using much higher concentrations of antibodies, or using high salt solutions to reduce the RNases activities (Grimm et al., 2004; Brown and Smith 2009; Tangrea et al., 2011a; Nichterwitz et al., 2016; Baccin et al., 2020). However, neither of these methods has proven sufficiently robust with moderate-to-high RNase-content tissues in terms of both high quality staining and high RNA quality to enable comprehensive cell-type specific transcriptomic analyses.

In this method paper, we present a broadly applicable immuno-LCM-RNAseq method that enables high quality RNAseq with a variety of tissues for spatial transcriptome acquisition from sections of either snap-frozen or RNAlater preserved clinical tissues. The power of this approach is demonstrated here with an initial characterization of the differences in the transcriptional programs between the lacteal tip and main body cells in the mouse small intestine, which implicate several genes involved in sensing and responding to local environmental cues to stimulate intra-cellular signalling. We anticipate that this method will become indispensable in the acquisition of spatial transcriptomes of phenotype-defined cells in their native environment, such as developing embryos or aging tissues, to enable a thorough understanding of the intrinsic signalling networks that ultimately underlie tissue growth, functioning, and decline.

## Materials and Methods

### Overview of Immuno-LCM-RNAseq Method

**FIGURE 1:**
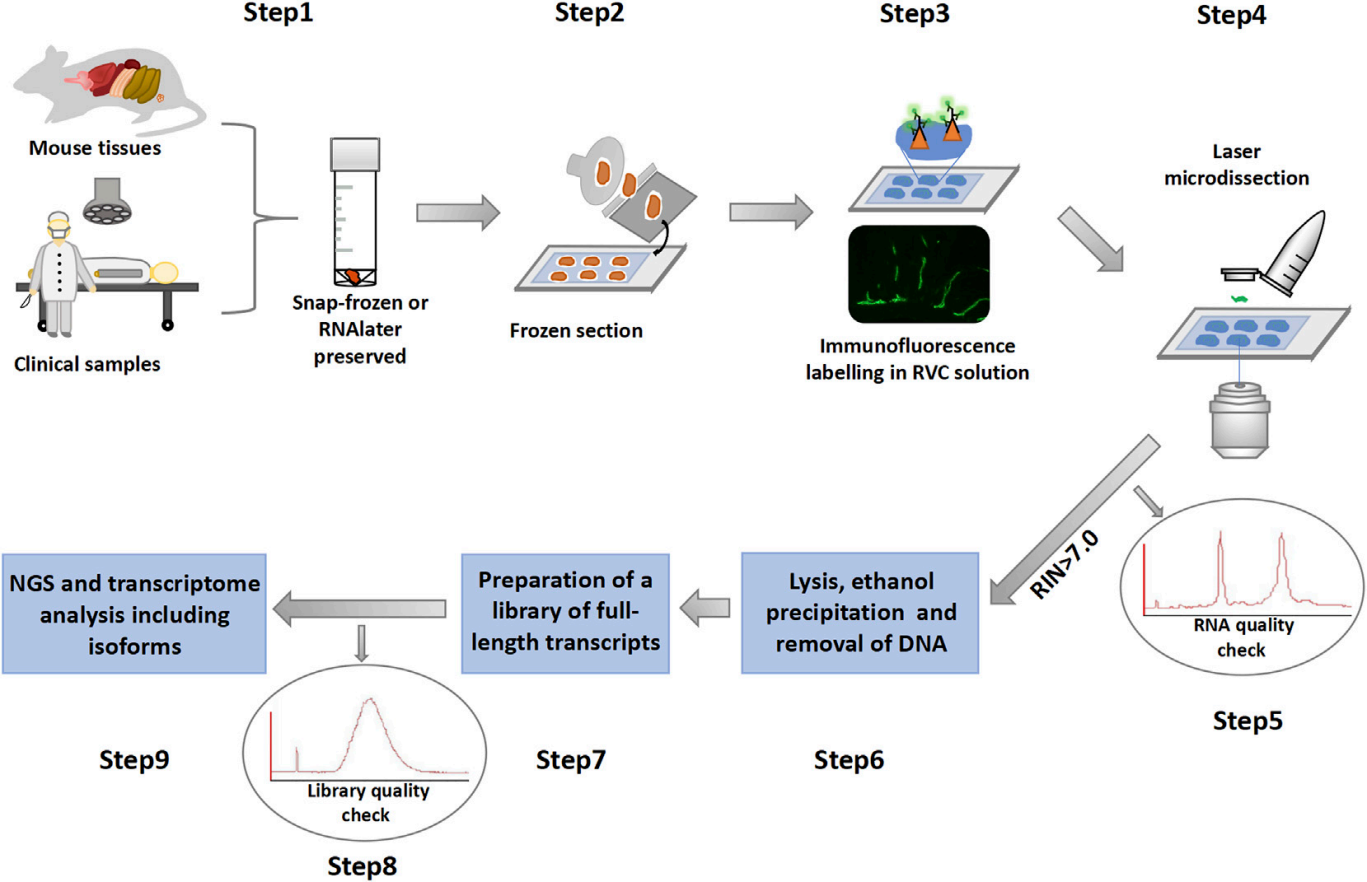
Overview of the immuno-LCM-RNAseq method.

The workflow of the immuno-LCM-RNAseq method consists of the following steps that are schematically presented in Figure 1:

**(1)** Tissue preservation with either snap-freezing or RNAlater treatment.
**(2)** Preparation of tissue sections in a cryostat at appropriate temperatures.
**(3)** After fixation and proper dehydration, standard two step immunostaining in a specially prepared solution that includes the small molecule RNase inhibitor (RVC, see below).
**(4)** Collection of immunofluorescence-defined cells with laser capture microdissection under controlled conditions.
**(5)** RNA quality check using leftover materials to determine whether to proceed.
**(6)** Extraction of RNA from collected cells to remove residual RNase inhibitor for subsequent steps.
**(7)** Preparation of cDNA libraries using the Ovation SoLo system (or its equivalent) for the analysis of full transcripts and isoforms.
**(8)** Quality check of the cDNA libraries with a 2100 Bioanalyzer before next generation sequencing.
**(9)** Construction of transcriptional programs and data analysis with publicly available software packages.

### Preparation of Snap-Frozen Tissues

6–8 weeks old C57BL/6 mice (Jie Si Jie Laboratory Animals, Shanghai, China) were used in these experiments. All mice were euthanized by cervical dislocation. The stomach, small intestine, liver, kidney, colon, spleen and testis were freshly dissected and cleaned in ice-cold RNase-free phosphate-buffered saline (PBS) solution. After absorption of excess liquid, tissues were placed in 2.5 ml capped cryogenic vials individually, and sealed with Parafilm (M2 Scientifics, cat. no. HS234526BC, Holland, Michigan, United States). Tissues were then snap-frozen in liquid nitrogen for 30 min and stored at −80°C.

### Sectioning of Snap-Frozen Tissues With the Cryostat

For snap-frozen tissues, the cryostat (Leica, cat. no. CM3050S, Buffalo Grove, Illinois, United States) was first defrosted and spray-cleaned with RNaseZap (Ambion, cat. no. AM9780, Austin, Texas, United States) and pure ethanol (Sigma-Aldrich, cat. no. E7023, St. Louis, Missouri, United States). The cryochamber temperature (CT) was set at -22°C and the specimen temperature (OT) was set at −20°C as commonly recommended (Dey 2018). The Optimal Cutting Temperature compound (OCT) (Agar Scientific, cat. no. AGR1180, Stansted, United Kingdom) was placed on ice for at least 30 min before use. A PET (polyethylene terephthalate)-membrane covered slide (Carl Zeiss, cat. no. 415190-9051-000, Jena, Germany) was cleaned with RNaseZap followed by UV irradiation for 30 min prior to mounting the sections. Most tissues were embedded in OCT and cut into 12 μm-thick sections, and mounted on PET slides. Sections on PET slides were dried for 2–3 min in the cryostat before fixation (for IF). For the mouse small intestine, it was critical to properly orient the tissue in the OCT in order to obtain desired sections. To this end, a frozen OCT block was first cut to obtain a flat surface, taking note of the cutting direction. The intestine tissue was then re-embedded on this flat surface with OCT, cut into 12 μm-thick serial sections and mounted on a PET slide.

### Immunostaining With the Rapid Protocol and the High-Salt Protocol

All immunostaining procedures were performed in a RNA-specific biological safety cabinet which was pre-cleaned by RNaseZap. However, antibody labelling was carried out at 4°C in a refrigerated chamber.

For the Rapid protocol, we followed a previously established procedure (Nichterwitz et al., 2016). The air-dried (in the cryostat) PET slide with the sections was fixed for 5 min in ice-cold acetone (Sigma-Aldrich, cat. no. 179124, St. Louis, Missouri, United States) followed by 3 quick washes (1 min) with RNase-free ice-cold PBS solution. The slide was then incubated with rabbit anti-mouse Lyve1 primary antibody (1:25, AngioBio cat. no. 11-034, San Diego, California, United States) in cold PBS with 0.25% Triton X-100 (Sigma Aldrich, cat. no. 93426, St. Louis, Missouri, United States) for 5 min at 4°C. After 3 quick washes with ice-cold PBS (1 min), the slide was incubated with secondary antibody (1:25; Alexa Fluor 488 conjugated goat-anti-rabbit; Invitrogen, cat. no. A11034, Carlsbad, California, United States) in ice-cold PBS with 0.25% Triton X-100 for 5 min at 4°C and then washed 3 times in ice-cold PBS (1 min).

For the high-salt protocol, we closely followed the original protocol presented in the reference (Brown and Smith 2009). In short, the sections were fixed in 70% ethanol for 5 min, then followed with a rapid PBS wash. Sections were incubated with rabbit anti-mouse Lyve1 antibody (1:100, AngioBio cat. no. 11-034, San Diego, California, United States) with 2 M NaCl in PBS overnight at 4°C. Unbound primary antibody was removed by 3 quick washes with 2 M NaCl in PBS for 5 min. Sections were then incubated with Alexa Fluor 488 conjugated goat-anti-rabbit (1:100, Invitrogen, cat. no. A11034, Carlsbad, California, United States) in 2 M NaCl PBS in the dark for 1 h at 4°C. Slides were then washed 3 times in 2 M NaCl PBS for 5 min. All of these solutions were ice-cold. We note that the original procedure used overnight incubation with the antibodies. Owing to the serious structural damage on the small intestine sections under this condition (see Supplementary Figure S1), we also examined a shorter incubation procedure similar to that described above except with a ∼3.5 h primary antibody labelling (∼5 h total incubation time). To reduce cross-reactivity, we also examined a procedure that included a blocking step, prior to incubation with the primary antibody, using a mixture of equal volume of ready-to-use protein block serum-free solution (Agilent Dako, cat. no. X0909, Santa Clara, California, United States) and 4 M NaCl in PBS at 4°C for 15 min followed by 3 quick washes with 2 M NaCl in PBS. In those experiments with this blocking step, we also diluted the antibodies in the 2 M NaCl solution and a 1:4 dilution of the ready-to-use protein block serum-free solution.

### Preparation and Sectioning of RNAlater Samples

For samples preserved in RNAlater (Invitrogen, cat. no. 7021, Carlsbad, California, United States) which protects RNA from degradation, tissues were first cut into pieces smaller than 0.5 × 0.5 × 0.5 cm^3^ and washed in ice-cold RNAlater solution, then left in the RNAlater solution at a RNAlater:sample volume ratio ≥10:1 at 4°C for 4–8 h. Excess RNAlater solution was then removed and the treated tissue was stored at −80°C. All experiments with animals were performed in compliance with the guidelines of the Institutional Animal Care and Use Committee of the Shanghai Jiao Tong University. The clinical human jejunum tissue was collected from a gastrectomy patient (Renji Hospital, Shanghai, China). The tissue was cut into small pieces and immediately placed into the RNAlater solution on ice. Tissue samples were stored at −80°C until use. Approvals were obtained from the Research Ethics Committee at Renji Hospital, Shanghai, China.

For RNAlater-preserved tissues, the CT and OT were kept below −28°C. This was achieved by flowing a stream of liquid nitrogen gas across the cryostat knife holder. The frozen RNAlater-preserved tissue often contained a thin layer of solidified RNAlater material on the surface, which prevented direct contact between the tissue and OCT, and often led to difficulties in proper sectioning. To remove this solidified RNAlater layer, we partially immersed the frozen RNAlater-preserved tissue in fresh ice-cold OCT, and after OCT solidified, removed the sample from the frozen OCT, which left a large portion of the RNAlater layer attached to the frozen OCT. This was performed repeatedly until the residual RNAlater layer was completely removed from the tissue. The tissue was then fully embedded within ice-cold OCT, cut into 12 μm-thick serial sections and mounted on a PET slide. The sections were dried for 2–3 min in the cryostat before fixation (for IF).

### Ribonucleoside Vanadyl Complex-Based Immunofluorescence Staining Procedure

The sections on PET slides were first fixed with cold acetone in the cryostat: 30 s for snap-frozen tissues and 5 s for RNAlater-preserved tissues. For the snap-frozen tissue, longer times (from 1 to 5 min) led to decreased RNA yields, whereas shorter times (5 s) led to insufficient fixation of the tissue. For the RNAlater samples, we found that a fixation time of 5 s led to a greater yield of RNA and antibody labelling than a 30 s fixation, likely owing to some level of fixation produced by the RNAlater solution (Páska et al., 2004; Hentze et al., 2019). The fixed sections were then dried in the cryostat for 5 min, and washed 3 times with ice-cold 10 mM Ribonucleoside Vanadyl Complex (RVC) (New England BioLabs, cat. no. S1402S, Ipswich, Mass, United States) in buffer A (10 mM NaCl, 3 mM MgCl_2_, 20 mM Tris•HCl, pH 7.4) in a RNA-specific biological safety cabinet. We refer to this 10 mM RVC solution as the RVC solution unless otherwise indicated. The sections were pre-blocked with an equal volume mixture of 20 mM RVC in buffer A and the ready-to-use protein block serum-free solution at 4°C for 15 min, followed by 3 times wash with the RVC solution. The slides were then incubated for 3.5 h with the primary antibody: either anti-mouse Pan Cytokeratin antibody (PanCK) (1:100, Santa Cruz Biotechnology, cat. no. sc-8018, Dallas, Texas, United States), anti-mouse Lyve1 antibody (1:100, AngioBio cat. no. 11-034, San Diego, California, United States) or anti-human Podoplanin antibody (1:100, ReliaTech, cat. no. 101-M41, Wolfenbuettel, Germany). After washing 3 times with the RVC solution, the slides were incubated with the secondary antibody, either Alexa Fluor 488 conjugated goat-anti-rabbit or donkey anti-mouse (1:100, Invitrogen, cat. no. A11034, A32766 Carlsbad, California, United States) or Fluor 568 conjugated donkey anti-mouse (1:100, Invitrogen, cat. no. A10037), in the dark for 1 h at 4°C. All antibodies were pre-diluted in the RVC solution and a 1:4 dilution of the ready-to-use protein block serum-free solution before use. After secondary antibody incubation, the slides were washed 10 times with the RVC solution and temporarily stored in a light-tight box until laser microdissection. For validation of the lymphatic vessel location in the small intestine or stomach tissue sections, we further stained with Hoechst (1:1,000 in RVC solution, Invitrogen, cat. no. H3569, Carlsbad, California, United States) in the dark at 4°C for 10 min, then washed 10 times with the ice-cold RVC solution. For maximum RNA protection, the RVC solutions must be freshly prepared before use: long term storage, even at 4°C, can severely reduce its effectiveness.

### Laser Capture Microdissection of Immunofluorescence Identified Cells

Laser capture microdissection was performed with the Zeiss PALM MicroBeam LCM system (Zeiss Microimaging, Munich, Germany) housed in a Plexiglas housing. The fluorescence microscope of this system has a resolution of about 0.25 μm, while the laser cutting precision can reach down to 0.7 μm under the ×40 objective and 0.6 μm under the ×63 objective. As such, the system has a high enough resolution to select single cells, and even single nuclei (Guo et al., 2012). Desired cells were manually selected on the control screen based on their fluorescence signal. We note that the fading of the fluorescence is generally not a significant problem during the cell selection process, as the LCM system provides the option to manually freeze images on the screen and then turn off the excitation light. Thus during most of the cell selection period, the excitation light is off. Before microdissection, excess solution was removed with a pipette from the slide and the sections were allowed to dry fully on the PALM stage under controlled humidity (humidity <45%). The dissected materials with the UV laser were ejected into 0.2 ml adhesive cap tubes (Carl Zeiss, cat. no. 415190-9191-000, Jena, Germany). The tubes were quickly removed and taken to a Biological Safety Cabinet (Thermo Fisher, 1300 Series A2) that was pre-cleaned with RNaseZap. 30 μl of GITC lysis buffer (Invitrogen, cat. no. 15577-018, Carlsbad, California, United States) was added to the cap with gentle pipetting. The tubes were then sealed with Parafilm and vortexed several times, followed by incubation at 42°C for 30 min to improve RNA extraction. The tubes were then centrifuged at 20,800 g for 10 min at room temperature and stored at −80°C for later use.

### Assessment of RNA Quality

For all of the following, RNA was extracted using the RNeasy Micro Kit (QIAGEN, cat. no. 74004, Hilden, Germany) and examined with the 2100 Bioanalyzer (RNA6000 Pico Kit, Agilent, cat. no. 5067-1,513, Santa Clara, California, United States), which can detect as low as 200 pg/μl RNA. The RNA quality of the tissues was initially evaluated by extracting the total RNA from a few pieces of the sections of either the snap-frozen or RNAlater preserved tissues. Only those tissues with an RNA integrity number (RIN) greater than 9.0 were used. The RNA quality was also evaluated after microdissection by examining the leftover materials from the same section to make sure that there was no serious degradation during the procedure. Only those with RIN >7.0 were considered of sufficient quality for further analysis. As documented in literature, an RIN >6.5 is generally considered sufficient for transcriptomic analyses as lower RIN samples often result in the loss of library complexity (Romero et al., 2014). These leftover materials were collected by LCM and placed into 350 μl of RLT lysis buffer (RNeasy, Qiagen, including 1% *β*-Mercaptoethanol (β-Me)) followed by RNA extraction and examination with the 2100 Bioanalyzer.

### Preparation of the cDNA Library

The samples stored in the GITC lysis buffer were thawed on ice, pooled, and then additional GITC lysis buffer was added to bring the total volume to 200 μl. RNA/DNA was precipitated by incubation at −80°C for 2 h with 600 μl cold ethanol (Sigma-Aldrich, cat. no. E7023, St. Louis, Missouri, United States), 20 μl 3 M NaAc (Amresco, cat. no. 97062-812, Soren, Ohio, United States) and 1 μl Glycogen (Invitrogen, cat. no. R0551, Carlsbad, California, United States). The samples were then centrifuged for 30 min at 4°C and the precipitate was washed 3 times with 75% cold EtOH, followed by dissolution in 10 μl RNase-free water (including 2U/μl SUPERase• In™ RNase Inhibitor). The DNA was digested with HL-dsDNase in DNase buffer (NuGEN, cat. no. 0354, San Carlos, California, United States) at 39°C for 15 min. The cDNA library was constructed using the Ovation SoLo RNAseq Kit (NuGEN, cat. no. 0354, 0352, San Carlos, California, United States), according to the manufacturer’s instructions. The number of optimal PCR cycles was determined by qPCR following the manufacturer’s recommendations. The cDNA library quality was evaluated using the 2100 Bioanalyzer (DNA high sensitivity kit, Agilent, cat. no. 5067-4626, Santa Clara, California, United States).

### RNAseq and Data Analysis

The cDNA library was sequenced on the Illumina high-throughput sequencing platform with the 2 × 150 bp pair-end mode. Raw reads were first submitted to Cutadapt-1.16 (Martin 2011) (with parameters of-u 5 -max-n 0 --minimum-length 100) to remove the sequencing adapters. The first 5 bases of each read were removed according to the library construction protocol of the Ovation SoLo RNAseq Kit. Trimmomatic-0.35 (Bolger et al., 2014) (with parameters of PE SLIDINGWINDOW:3:10 LEADING:10 TRAILING:10 MINLEN:100) was employed to remove low quality reads. SortMeRNA-v2.1b (Kopylova et al., 2012) was used to remove rRNA reads in the pair-end mode with default parameters. The cleaned reads were manually inspected by the Q30 profile of FastQC-v0.11.5 (Andrews 2010) to ensure sufficient data quality for further analysis. For the mouse data, the cleaned reads were mapped to the mouse GRCm38 (mm10) genome assembly with hisat2-2.0.5 (Kim et al., 2015; Pertea et al., 2016) in a strand-specific manner (with parameters of--rna-strandness FR). For the human data, the cleaned reads were mapped to the human GRCh38 (hg38) genome assembly with hisat2-2.0.5 (Kim et al., 2015; Pertea et al., 2016) also in a strand-specific manner (with parameters of--rna-strandness FR).

To evaluate the reproducibility of the replicates, the mouse genome was partitioned into 1 kb bins and the number of clean reads in each bin was counted with bedtools (v2.27). The Pearson correlation coefficient was then calculated pairwise between the samples. Transcript and gene level expression was estimated with StringTie-1.3.3 (Pertea et al., 2015; Pertea et al., 2016) (with parameters of -e -b) based on the Ensembl gene model (Mus_musculus.GRCm38.94.gtf and Homo_sapiens.GRCh38.94.gtf). Uniquely mapped clean read counts were normalized into FPKM (fragments per kb per million) to quantify gene and transcript expression.

The correlation between mouse small intestine lacteal tip and tube cells was calculated by using the average gene expression levels of the combined data of all replicates for each. Differential expression was examined between the tube samples (4 replicates) and the tip samples (3 replicates) using the DESeq2 package (1.28.1) (Love et al., 2014) with default settings. A subprogram prepDE.py of StringTie was employed to derive hypothetical read counts for each gene or transcript and the derived reads count matrix served as the input file of DESeq2 to conduct differential analysis at both the gene and transcript isoform level. Genes or transcripts with a log_2_FoldChange >2 or log_2_FoldChange < -2 and p_adj_ < 0.01 were considered differentially expressed. Hierarchical analysis was performed by measuring the average Euclidean distance between different clusters. Gene body coverage plot was generated by RSeQC (v3.0.1) (Wang et al., 2012). Tube-specific highly expressed genes were classified according to GO analysis using the ShinyGo webserver (Ge et al., 2020).

For the analysis of the data from the recent LCM RNAseq study of bone marrow (Baccin et al., 2020), we randomly selected nine samples with ∼20 million reads (as examples with a comparable read depth as our small intestine epithelial samples) and four samples with ∼30 million reads (as examples with ∼50% greater read depth as our samples). Each sample was analysed using the same pipeline as our data (see above) except that parameters within sortMeRNA-v2.1b (Bolger et al., 2014) and hisat2-2.0.5 (Andrews 2010; Kim et al., 2015) were adjusted for processing of these 75 bp-single end datasets rather than our paired-end datasets.

## Results

### Evaluation of the Effectiveness of the Present Immunolabelling Methods for LCM-Seq

**FIGURE 2:**
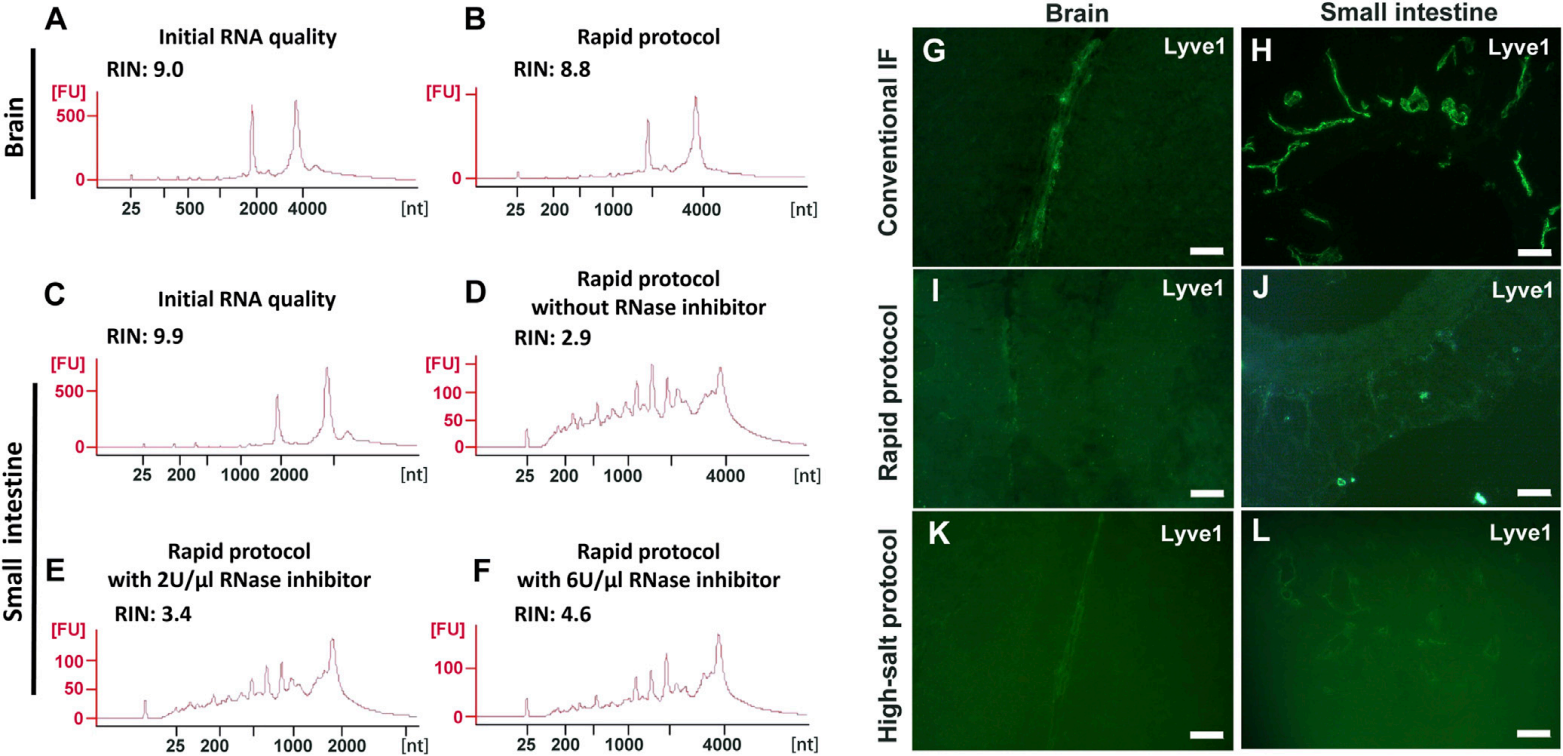
Evaluation of the Rapid and high-salt immunofluorescence staining protocols with frozen sections of the mouse brain and small intestine. **(A-F)** RNA quality assessment for frozen sections after immunofluorescence labelling using the Rapid protocol. For the brain sections, the RNA quality after rapid immunolabelling **(B)** is comparable to the initial quality **(A)**. For the small intestine with a high endogenous RNase content, the RNA were severely degraded **(D)** even when high concentrations of RNase inhibitor were present **(E–F)**. **(G-L)** Quality of IF images with the mouse brain **(G,I,K)** or the small intestine **(H,J,L)**. Here, anti-Lyve1 was used to identify lymphatic vessels. With both protocols **(I–L)**, the image quality in both tissues is poor when compared with the control **(G,H)**. Scale bar: 50 µm **(G,I,K,L)** and 100 µm **(H,J)**.

We first re-evaluated the image quality and extent of RNA protection of the existing approaches used for immuno-LCM, namely “Rapid Immunostaining” (hereafter called the “Rapid protocol”) (Nichterwitz et al., 2016) and using high concentrations of salt (the “high-salt protocol”) (Brown and Smith 2009), during immunostaining with snap-frozen tissues. The Rapid protocol entails the incubation of the sections with high concentrations of antibodies (1:10 to 1:25 dilutions, about ten-fold greater than that recommended for regular IF staining) in the presence of high concentrations of a potent RNase inhibitor (1–2 U/µl) (Grimm et al., 2004). The complete procedure, which typically requires a minimum of several hours with conventional IF staining (Im et al., 2019), is drastically shortened to only 3–15 min. The high-salt protocol involves the addition of 2 M NaCl to the antibody incubation solution but otherwise follows the conventional several-hour procedure to complete. Neither of these protocols recommended a pre-block step although such pre-blocking is known to reduce background in most immunolabelling procedures to improve image quality. Closely following these protocols, we examined their effectiveness to produce high quality fluorescence images and to preserve RNA quality using an anti-Lyve1 antibody (1:25 dilution, 40 μg/ml) to label lymphatic vessels in frozen sections of the mouse brain and small intestine. RNA quality was assessed with entire sections after the immunostaining step but without laser dissection. For the brain sections, which are largely devoid of endogenous RNases, both methods yielded good quality RNA with RIN values of ∼9.0 (Figures 2A–F and Supplementary Figure S1). However, the contrast (S/N) in the fluorescent images obtained with either method (using 15 min incubation for the Rapid protocol) was poor when compared to that obtained with the conventional protocol (Figure 2G-L and Supplementary Figure S1). In fact, such low quality images make unambiguous identification of targeted cells, a prerequisite for LCM, nearly impossible.

For the mouse small intestine, which has a much higher RNase content than the brain (Uhlén et al., 2015; Walker et al., 2016; https://www.proteinatlas.org/ENSG00000129538-RNASE1/tissue), only the high-salt protocol provided good RNA protection, albeit with a moderate degradation with increased incubation time (RIN: 8.6 at 5 h vs. 7.0 overnight) (Supplementary Figure S1). However, with this approach, not only was the IF image poor but the tissue structure was severely compromised (Figure 2L, Supplementary Figure S1). This structural degradation essentially prohibited any meaningful investigation of the spatial transcriptome as the section had lost many of its constituent cells. For the Rapid protocol, the procedure failed to provide effective RNA protection and the extracted RNA only had a RIN of 2.9. At the same time, the fluorescence image remained poor (Figure 2J). We note that this degradation of RNA with the Rapid protocol occurred despite the presence of recombinant RNase inhibitors (2 U/µl). With a further increase of the RNase inhibitors up to 6 U/µl (3-fold higher than the recommended value), the observed improvements were disappointing if at all (Figures 2E,F).

One possible reason for this failure of the RNA inhibitors to protect RNA in RNase-rich sections is that the diffusion of this inhibitor, a relatively large protein (∼50 KD with an estimated dimension of 7 × 6 × 3 nm^3^ (ref. Hofsteenge 1997)) (Supplementary Figure S2), might be too slow in tissue sections, as tissues are essentially dense, gel-like matrices (Davies et al., 2002). This might be a problem further exacerbated by the required dehydration-rehydration steps. Hence, it could be that it takes too long for the inhibitors to reach deep inside the section following rehydration, leading to rapid RNA degradation by the abundant endogenous RNases before the inhibitors arrive. As such, this type of RNase inhibitor is poorly suited for use with tissue sections.

### Development of a Strategy Using a Small Molecule RNase Inhibitor for Generally Effective Immuno-LCM-RNAseq

Given the ineffectiveness of the recombinant RNase inhibitors, we reasoned that small molecule RNase inhibitors might confer a much better protection owing to their faster diffusion within the rehydrated tissue section and thus quicker deactivation of the endogenous RNases. In this regard, it has been known that nucleoside analogues are potent inhibitors of many classes of nucleases (Russo et al., 2001). Among the many candidates, the ribonucleoside vanadyl complex (RVC) (Berger and Birkenmeier 1979) is particularly attractive since it is a transition-state analogue (Lienhard et al., 1972; Leon-Lai et al., 1996): these complexes specifically bind to the catalytic site of ribonuclease (Supplementary Figure S2) and should be broadly effective against many different RNases (Berger and Birkenmeier 1979). Although these complexes have been used to preserve RNA in tissues or during RNA *in situ* hybradization (RNA-FISH) in a few studies previously (Shieh et al., 2018; Credle et al., 2017; Lyubimova et al., 2013; Patel et al., 2021), they have been largely superseded by the more potent recombinant RNA inhibitors in most other experiments (Shapiro 2001; Dickson et al., 2005; Mita et al., 2021; Popella et al., 2021; Qian et al., 2020; Arizti-Sanz et al., 2020). Whether RVC could provide robust RNA protection following immunolabelling, or immuno-LCM, which typically require many hours to complete, has not been previously examined. To this end, to compare its effectiveness with the aforementioned approaches, we first examined mouse brain frozen sections during the standard long time immunostaining with the anti-Lyve1 antibody that identifies lymph vessels with different concentrations of RVC in the incubating solutions. As with the standard immunofluorescence staining, cooled acetone fixed brain sections were first blocked (to reduce non-specific binding) for 15 min, followed by incubation with the primary antibody (1:100 dilution as recommended; ∼10 μg/ml) for 3.5 h, followed by secondary antibody (10 μg/ml) incubation for 1 h at 4°C. We compared the results in 3 different RVC concentrations in all of the buffer solutions: 2.5, 5 or 10 mM. We found that RVC had negligible effects on antigen-antibody interactions at these concentrations. As shown in Supplementary Figure S3, the resultant IF images were of high contrast, essentially the same as those without the addition of any RVC. When the RNA quality of these treated sections was assessed, we found that samples with 5 mM or 10 mM RVC provided superb RNA protection with RIN >9.5. But at 2.5 mM RVC, some RNA degradation was apparent (RIN 7.2) (Supplementary Figure S4). These results demonstrate that, at least for brain sections, a minimum of 5 mM RVC is required for conventional (high quality) immunostaining to ensure fully protected RNA. We should also indicate that, in the process of performing these experiments, we found that the potency of the RVC solutions to protect RNA decreased over the course of days (similar to previous reports) (Berger and Birkenmeier 1979). However, use of freshly prepared RVC solutions proved to be a simple, effective remedy of this problem.

**FIGURE 3:**
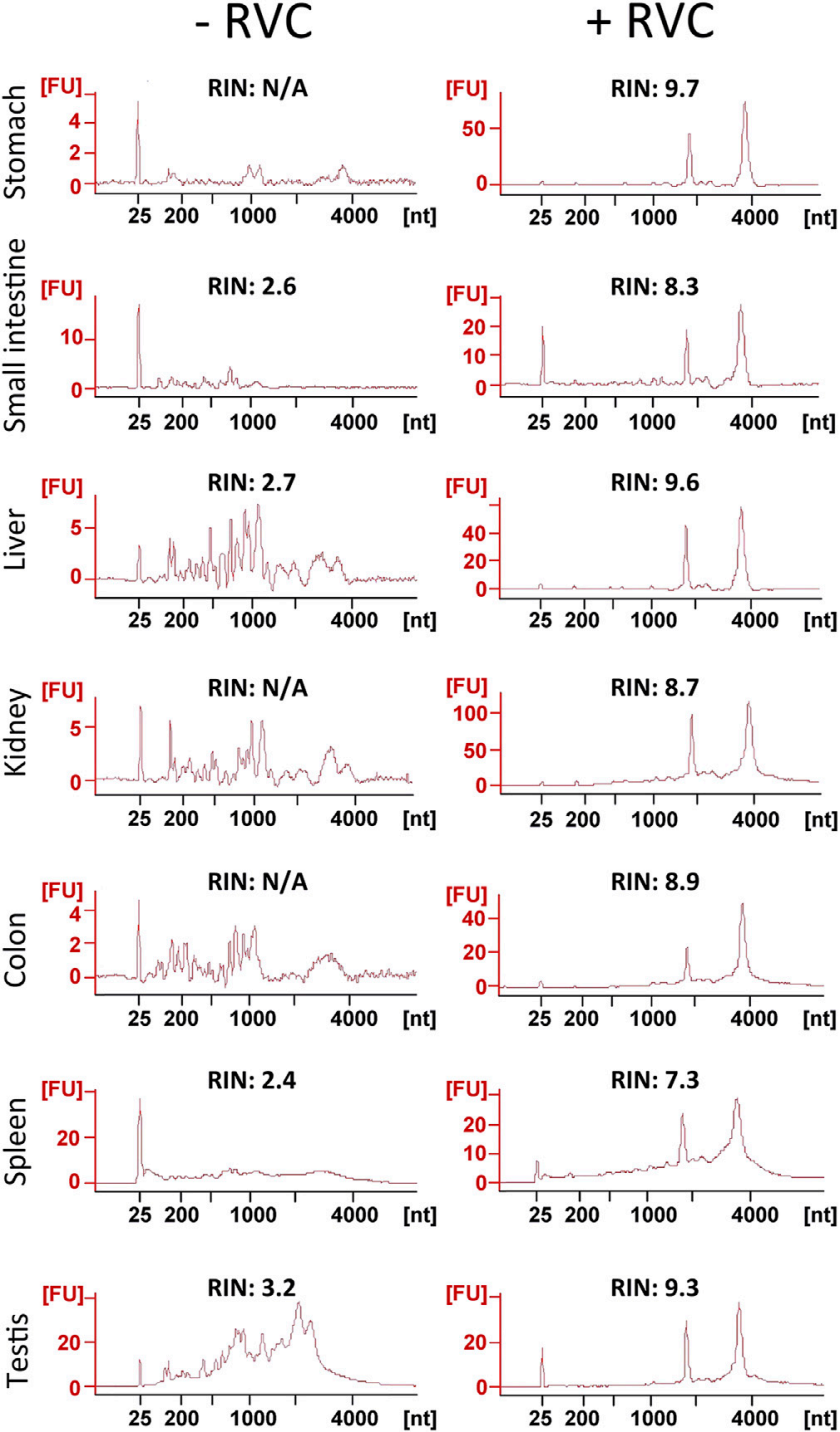
Assessment of RNA quality from sections of various snap-frozen mouse tissues after the standard immunolabelling procedure (details in the text) in the presence or absence of 10 mM RVC. As shown by the RIN values, RVC provided superb RNA protection in all of these RNase-rich tissues during the lengthy immunolabelling procedure.

**FIGURE 4:**
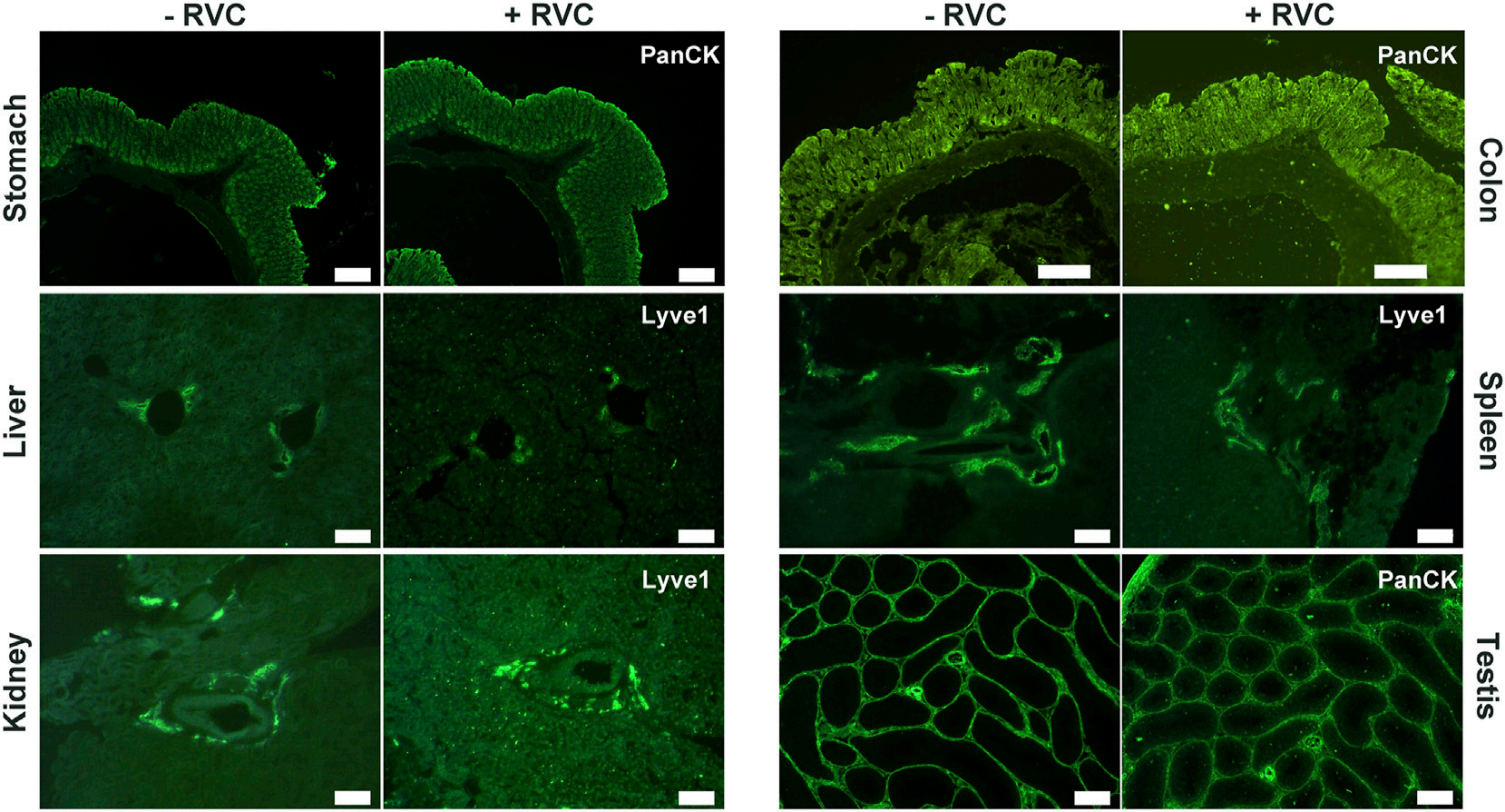
High quality IF images obtained from various snap-frozen tissue sections with standard immunolabelling procedures in the presence of 10 mM RVC. The presence of RVC in the incubation solution apparently has no adverse effect on antibody-antigen interactions. Here, anti-Lyve1 labels lymphatic vessels and anti-PanCK labels epithelial cells. Scale bar: 200 μm (stomach, colon, testis), 100 μm (liver, kidney, spleen).

We next examined the effectiveness of RVC with other frozen tissue sections containing moderate-to-high levels of RNase (Uhlén et al., 2015; Walker et al., 2016; https://www.proteinatlas.org/ENSG00000129538-RNASE1/tissue). With the mouse small intestine, we found that 5 mM RVC was not sufficient to fully protect the RNA (RIN 6.3), but with 10 mM RVC, the quality of RNA was significantly improved (RIN 8.1) while the IF images remained excellent (Supplementary Figures S3, S4). Further increasing the RVC concentration (up to 20 mM) resulted in a moderate improvement in RNA quality (RIN 8.8) but with some adverse effects on the antibody-antigen interactions, leading to a deterioration of the IF images (Supplementary Figures S3, S4). Therefore, we used 10 mM RVC as the optimal working concentration to examine its protective effect with various mouse frozen tissue sections: the stomach, liver, kidney, colon, spleen and testis (Figure 3). For each of these tissues, and for all antibodies tested so far, 10 mM RVC invariably provided robust RNA protection with RIN values ranging from 7.3 (spleen) to 9.7 (stomach) (Figure 3). When RVC was absent, the best RNA quality that could be obtained with these tissues was around RIN 3.2 (testis), far below that required for high quality RNA profiling. Similar to the mouse brain sections, high quality IF images were obtained with all of these tissues at 10 mM RVC (Figure 4). Such a high quality is more than adequate for precise microdissection.

**FIGURE 5:**
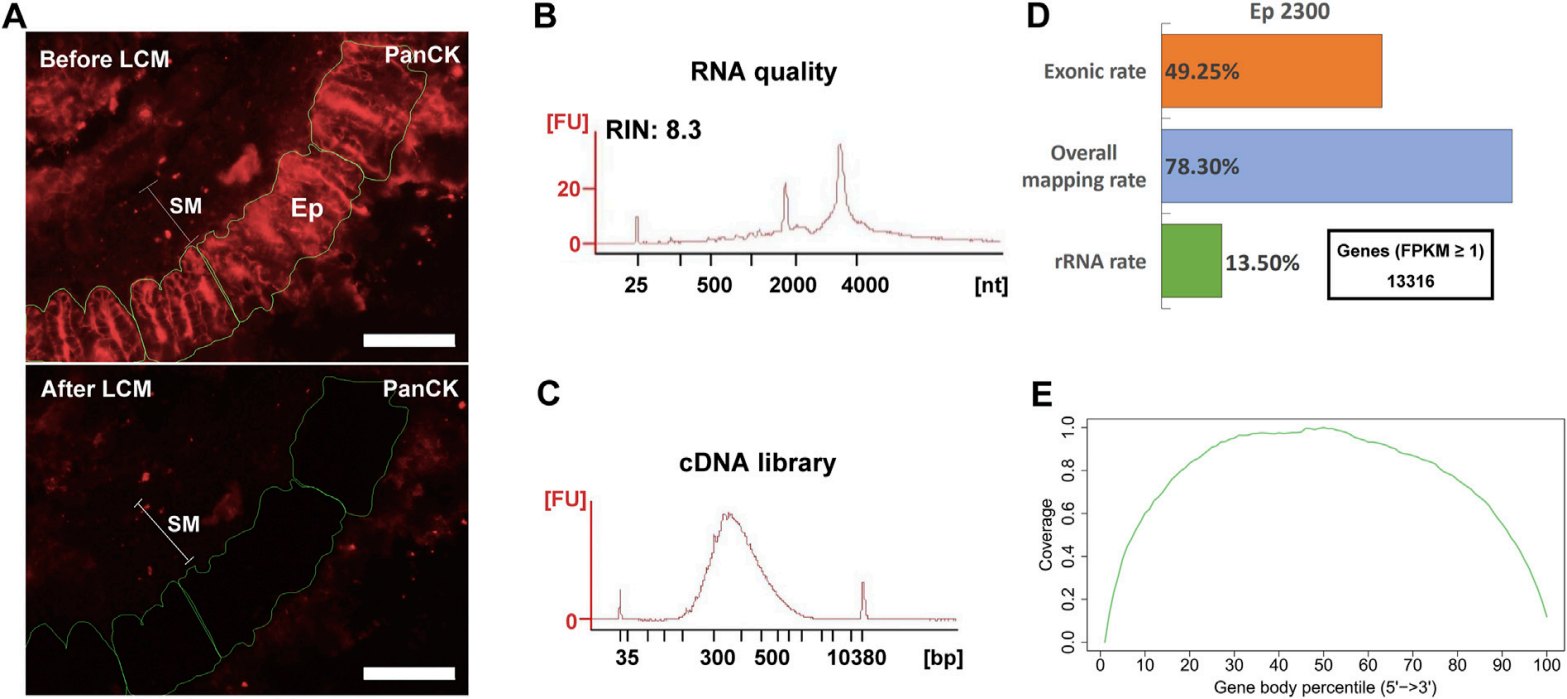
Immumo-LCM-RNAseq with ∼2300 cytokeratin positive epithelial cells (Ep2300). **(A)** A typical anti-PanCK stained fluorescence section before (upper) and after (lower) laser microdissection. SM: submucosal layer; Ep: cytokeratin positive cells. Scale bar: 100 μm. An image showing the nuclei stained with Hoechst is shown in Supplementary Figure S6. **(B)** High RNA quality of the leftover materials after microdissection. **(C)** cDNA library quality assessment. **(B)** and **(C)** were obtained with the 2100 Bioanalyzer. **(D)** Summary of the sequencing data quality with more than 13,000 expressed genes detected. **(E)** As expected from the Ovation SoLo system, normal reads coverage over the transcripts was obtained without 3′ enrichment. This coverage is required for full transcript and isoform analysis.

Based on the above findings, we finally examined the efficacy of the complete LCM-RNAseq procedure with IF-based cell identification, incorporating 10 mM RVC in all incubation/wash steps (immuno-LCM-RNAseq; see Figure 1 for the protocol flow chart). From sections of the mouse small intestine, the PanCK antibody was used to identify epithelial cells (Figure 5A, upper panel). The identified target cells were then manually marked and automatically laser dissected and collected with the Zeiss PALM MicroBeam LCM system. We note that the decrease in fluorescence intensity evident after LCM in Figure 5A occurs consequent to the drying of the sample and not during the cell selection process itself (see Methods), and so generally does not limit the number of cells that can be isolated in this experiment. The dissection and collection process were completed within 45–60 min at room temperature. This length of time was found to be optimal for the maximal number of cells that can be collected in one experiment with no significant decrement of the RNA quality, the latter of which tended to worsen with time during this step. As a preliminary test, we microdissected about 2,300 cytokeratin-positive epithelial cells (Ep2300) from the crypt region (within 100 μm from the submucosa) of 12 μm thick sections of the small intestine (Figure 5A, Supplementary Figure S5, S6; Supplementary Table S1). Examining material leftover after the laser microdissection, we found that the RNA integrity was sufficiently retained (RIN 8.3) (Figure 5B). Since the dissection was performed under ambient conditions in open air where a small amount of water is condensed on hydrophilic surfaces (Clement-Ziza et al., 2008), such robust RNA protection suggests that the residual RVC after section dehydration (as necessary for laser microdissection) remained effective against airborne RNase contaminants (Clement-Ziza et al., 2008; Bath et al., 2014).

One problem with the use of RVC is its effect on downstream procedures, including reverse transcription and/or PCR that are required for the construction of cDNA sequencing libraries, owing to its adverse effect on polymerases (Lau et al., 1993) (Step 7, Figure 1). Hence, RVC must be removed from the dissected materials before the downstream procedures can be performed properly. We used the ethanol precipitation/extraction method to purify RNA for the downstream construction of the cDNA library (removing residual DNA by DNase digestion) in this protocol. For the Ovation SoLo RNAseq system, which is optimized for low input RNA down to 10 pg and also adequate for mRNA transcript isoform analysis, the purified RNA was sufficient to construct the cDNA library (Nguyen et al., 2018). As shown in Figure 5C, the cDNA library obtained is indeed of high quality with a proper fragment distribution. After sequencing, we obtained about 16 million clean reads for this sample (Supplementary Table S2). The overall mapping rate was 78% and the exonic rate was 49%, both consistent with the expected outcomes of the Ovation SoLo system (Figure 5D). The rRNA contamination rate is 13.5% (Figure 5D), which is typical (15–20%) for library construction methods using NuGen random and oligo-dT mixed primers (Chao et al., 2019). Using FPKM ≥1 as the threshold, more than 13,000 expressed genes were identified (Figure 5D). The reads coverage across the gene body showed no bias towards the 5′ or 3’ end (Figure 5E), an indication of high quality RNA (Wang et al., 2016). Such a quality should be appropriate to investigate full-length transcripts and isoforms. Thus, these results demonstrate the effectiveness of our immuno-LCM-RNAseq protocol to obtain high quality transcriptomes (Figure 1).

### Determining the Working Limit of Immuno-LCM-RNAseq

**FIGURE 6:**
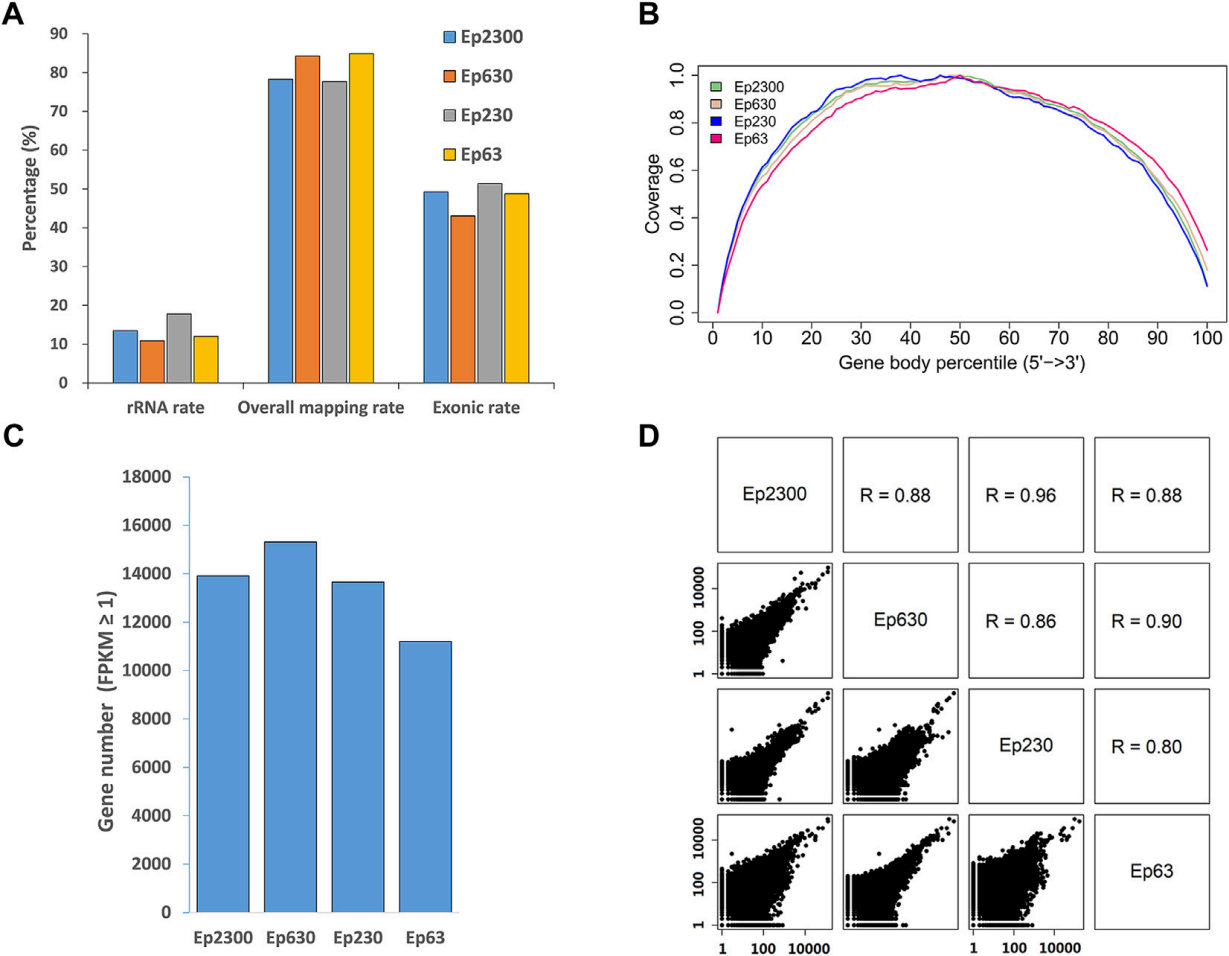
Assessment of the data quality and reproducibility with different amounts of input material dissected from the mouse small intestine using PanCK as the marker. The number of cells in each sample is indicated by the value following “Ep”, i.e., Ep63 = 63 cells. **(A)** Proportion of the overall mapping rate, the rRNA mapping rate and exonic rate of the samples. Despite a greater than 30-fold difference in the input material, the data quality remained consistent. **(B)** Reads distribution over the gene body is normal, without 5′ or 3′ end enrichment. **(C)** The number of expressed genes detected in all of the samples is similar, but is a little lower with the Ep63 sample, suggesting that the RNA extraction step could be further optimized. **(D)** Pairwise scatter plots between all samples. These Pearson correlation coefficients indicate that these results are consistent with each other.

Since any RNA extraction procedure will lead to a certain amount of material loss, we sought to determine the minimal number of dissected cells required for successful immuno-LCM-RNAseq. Again, using the PanCK antibody to identify the epithelial cells in the snap-frozen mouse small intestine sections, we laser dissected 630, 230, or 63 cells, also in the crypt region. Together with the Ep2300, we evaluated the consistency between the obtained transcriptomes of these samples (Figure 6) at a comparable level of sequencing depth (13–20 million clean reads) (Supplementary Table S2). Despite the significant difference in the amount of material collected (>30 fold), a similar number of expressed genes were identified under the same threshold (FPKM ≥1) (Figure 6C), demonstrating a high efficiency of this method. The reads coverage across the gene body also shows no bias towards the 5′ or 3’ end (Figure 6B). However, we noted that at the low end of ∼60 cells, the number of detected genes is slightly lower than the other samples, most likely owing to material loss in the RNA purification step. Nonetheless, the Pearson correlation (R) among these samples remained high (1 kb bin) (average R = 0.88) (Figure 6D), demonstrating the reproducibility over a large range of collected input materials. Therefore, our current protocol with ethanol precipitation combined with the Ovation SoLo system remains robust down to a few tens of dissected cells.

This analysis also enables a comparison with results obtained in a recent LCM-RNAseq study of bone marrow tissue (that has a similar level of RNase content as the small intestine (Uhlén et al., 2015; https://www.proteinatlas.org/ENSG00000129538-RNASE1/tissue; https://www.proteinatlas.org/ENSG00000169385-RNASE2/tissue)) in which the Rapid protocol was used during immuno-labelling. Although the main focus of this work was the characterization of the transcriptomic details of mixtures of 200-300 cells in different niches, and not a comprehensive understanding of phenotype-specific cells, their results serve a useful indicator of what can be presently achieved with the Rapid protocol. From a comparable number of cells, and at a similar sequencing depth, we were able to identify more genes (13521 vs. 3266), with a lower rRNA mapping rate (14 vs. 65%) and a lower intergenic mapping rate (7 vs. 15%), as well as superior overall and exonic mapping rates (Supplementary Table S2), with our approach. Thus, while this Rapid protocol method can provide useful information about the cellular organization of tissues, our method enables a more comprehensive understanding of the spatial transcriptome.

### Spatially Defined Expression: Comparing the Cells at the Tip and in the Main Capillary Body of the Lacteal

**FIGURE 7:**
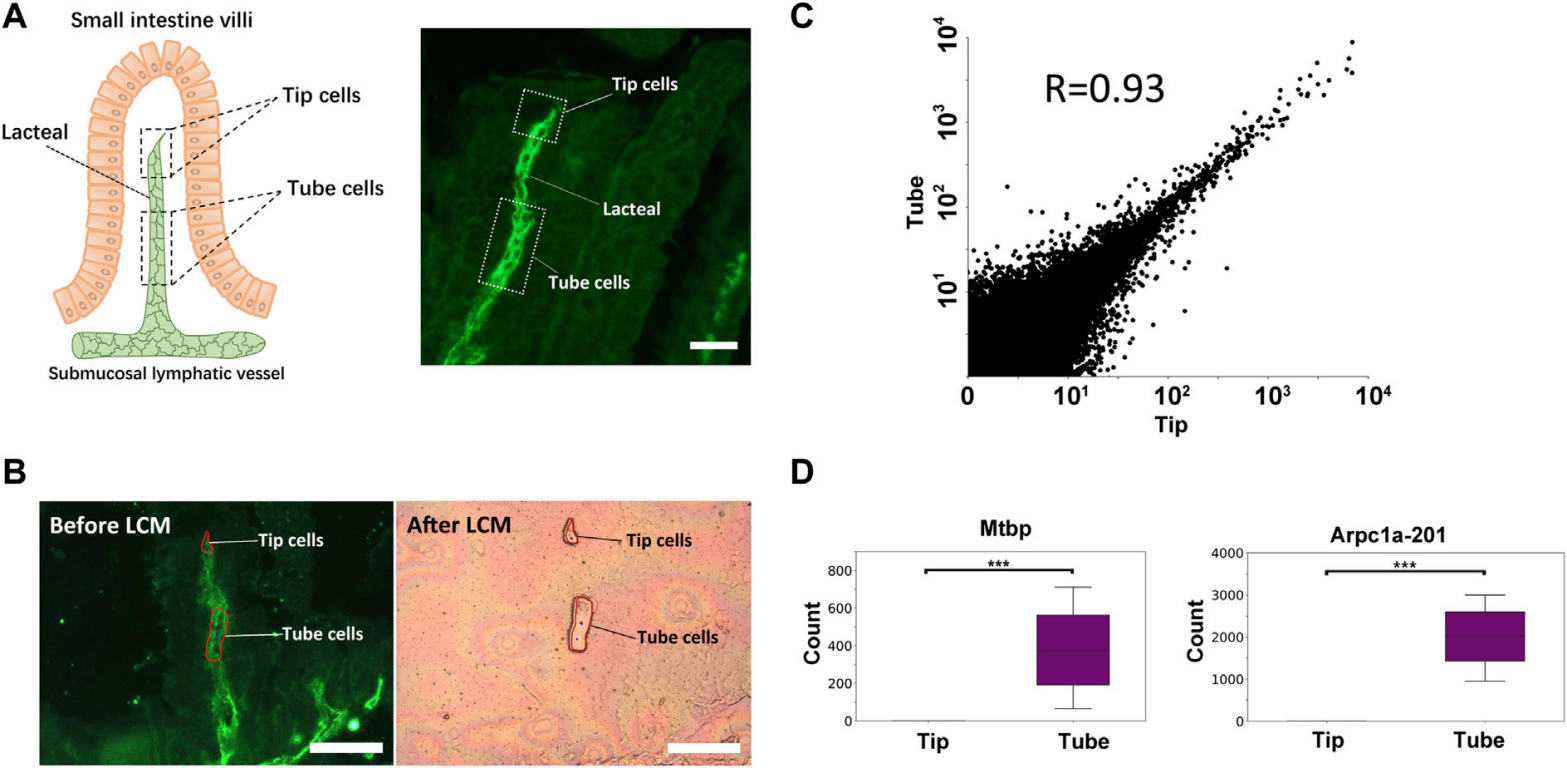
Transcriptome differences between the tip and the tube of the mouse small intestine lacteal. **(A)** Illustration of the small intestine lacteal structure (left) and a typical IF image of the lacteal (right), labelled with an anti-Lyve1 antibody. Scale bar: 50 μm. **(B)** Microdissection of the lacteal: tip cells and tube cells are collected separately with LCM. Each tip sample was pooled from up to 100 lacteals. Scale bar: 100 μm. **(C)** Scatter plot of the expression levels of the tip and the tube with all replicates combined. The highly consistent nature of the transcriptome is expected since these are all lymphatic endothelial cells. Although both the tip and the tube are considered to have the same phenotype, significant expression differences are still identified. Shown in **(D)** are two genes that are only expressed in the main body of the tube: *Mtbp* and the *Arpc1a*-*201* isoform (***, p_adj_ < 10^−5^).

We next applied immuno-LCM-RNAseq to examine an interesting fine structure, namely, the mouse small intestine lacteal, which cannot be readily resolved based on cell morphology alone (Supplementary Figure S7). The lacteal is the lymphatic capillary in the small intestine villi with crucial roles in fat absorption and gut immune response (Bernier-Latmani and Petrova 2017). However, unlike other lymphatic vessels, the lacteal cells are found to be moderately proliferative and exhibit long filopodia-like protrusions at the lacteal end (the “tip” cells) (Bernier-Latmani and Petrova 2017). As shown in Figure 7A, these fine capillaries can be clearly identified with the anti-Lyve1 antibody. We first dissected and collected ∼150 Lyve1 positive cells from the main body of the lacteal (50–70 μm away from the lacteal tip, referred to as the “tube”) (Figures 7A,B, Supplementary Table S2, S3) with excellent RNA quality (RIN 8.7, Supplementary Figure S8). With a total of 4 replicates, we obtained 49–63 million clean reads for each sample and the average overall mapping rate was 80%. The average Pearson correlation coefficient between all pairwise comparisons was 0.86. On average, more than 11,000 expressed genes were detected in each sample with FPKM ≥1 (Supplementary Table S2).

We then dissected a minute amount of material from the tip of the lacteals (∼20 μm in length, equivalent to one to two cells). We pooled this material into three independent replicates, each with an estimated 150 cells. We obtained 50–58 million clean reads for each and the average overall mapping rate was 78% (Supplementary Table S2). As expected, the averaged Pearson coefficient between pairwise comparisons is 0.89 (Supplementary Figure S9). Using FPKM ≥1, about 12,000 expressed genes were detected for each sample (Supplementary Table S2).

Combining all replicates, we explored the possibility of identifying differentially expressed genes or transcript isoforms using the program DESeq2. Despite the fact that the overall transcriptomes of the tip and the tube are highly similar (R = 0.93; Figure 7C), which was expected since they are both lymphatic endothelial cells, DESeq2 was still able to identify several genes and transcript isoforms with a statistically significant difference in expression (Supplementary Figure S10, Supplementary Table S5). Among these, the genes *Tnfsf15*, an established activator of lymphatic endothelial cell growth (Qin et al., 2015) and *Slfn1*, which inhibits the proliferation and tube formation of endothelial progenitor cells (Bragy et al., 2005; Kuang et al., 2014), were found to be only expressed in the tip of the lacteal (Supplementary Table S5), suggesting that the balance of these two proliferation-related genes could contribute to maintenance of the tip length. In addition, *Mtbp* and the protein-coding isoform of *Arpc1a* (201), both encoding for actin-filament severing proteins, were not expressed in the tip of the lacteal but robustly expressed in the tube cells (Figure 7D, Supplementary Table S4, S5) (Agarwal et al., 2013; Abella et al., 2015). *Arpc1a* is highly conserved and its protein product is one of the components of the Arp2/3 complex that plays an essential role in generating branched actin filaments. The loss of *Arpc1a* was reported to result in long actin tails both in cells and *in vitro* (Abella et al., 2015), and thus, could be related to the presence of long filopodia-like protrusions at the lacteal tip.

Additionally, we found that many of the highly expressed genes in the lacteal tube clustered in functional groups defined by high-level GO terms that include establishment of localization, regulation of signaling, and response to external stimulus (Supplementary Table S6). It is interesting to note that with the latter groups, genes that are upregulated include the well-known MAP kinase, *Map2k5*, the homeodomain interacting protein kinase, *Hipk4*, and *Rita1*, a tubulin-binding protein that acts as a negative regulator of Notch signaling, suggesting that these genes might play an important role in mediating signal transduction specifically in the tube cells, as well as *Kcnn4*, a voltage-independent potassium channel regulated by extracellular calcium that could play a critical role in sensing and responding to changes in the extracellular milieu (Kamakura et al., 1999; Pearson et al., 2001; Grgic et al., 2009; He et al., 2010; Wacker et al., 2010). Although further work is needed to fully characterize the functional consequences of these differences in expression, these results provide a clear example of the power of spatially-resolved transcriptional analysis to provide insight into potential mechanisms underlying cell functionality within complex tissues. It should also be noted that only with transcript isoform analysis could the difference in *Arpc1a*-*201* expression in particular be detected with certainty for which high RNA quality is paramount.

### Extension of Immuno-LCM-RNAseq to RNAlater-Preserved Tissues

**FIGURE 8:**
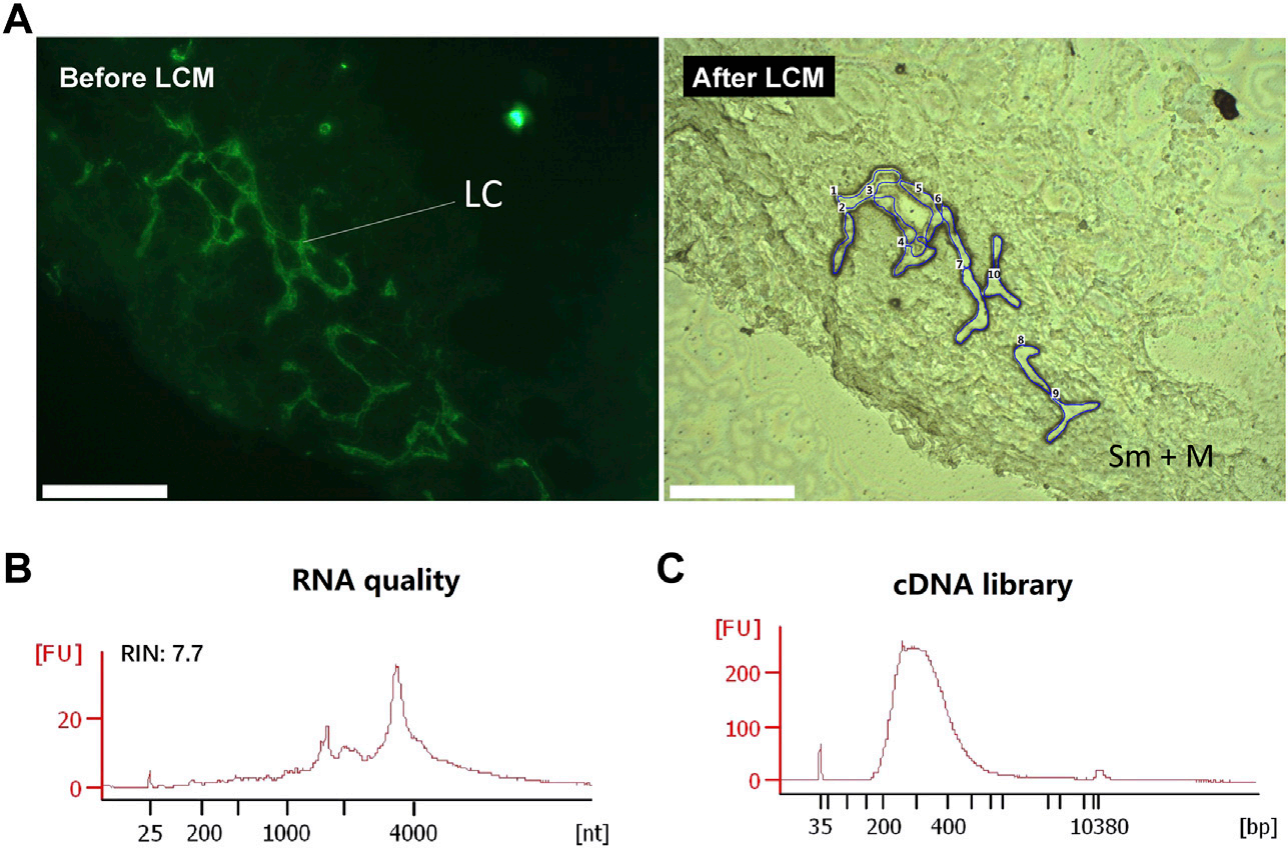
Immuno-LCM-RNAseq of the human jejunum lymphatic vessel from a clinical, RNAlater-preserved jejunum tissue. **(A)** Left: Immunofluorescence image of the lymphatic vessels in the human jejunum tissue section using an anti-Podoplanin antibody (with 10 mM RVC). Right: bright field image after LCM. LC: lymphatic cells; Sm: submucosal layer; M: muscle layer **(B)** RNA quality of the leftover materials after LCM. **(C)** The quality of the cDNA library prepared from this RNA. Scale bar: 200 μm.

While snap-freezing is the preservation method-of-choice for biological research, clinical tissues are often preserved with RNAlater owing to its convenience and potency to protect RNA during long-term storage at cryogenic temperatures (Florell et al., 2001; Mutter et al., 2004; Kasahara et al., 2006; Diaz et al., 2013). To enable full use of these resources, we further sought to extend our method to the tissues preserved with RNAlater (Figure 8). However, one of the often encountered problems with RNAlater preserved tissues is a difficulty to section properly in a cryostat (Ellis et al., 2002; Legres et al., 2014). We found that this difficulty largely stemmed from the softness of the treated tissue at typical cryostat cutting temperatures (−20°C). We found that at ∼ −24°C, the RNAlater solution alone freezes and stiffens significantly. Hence, by lowering the cutting temperature to ∼ −28°C by externally introducing streaming liquid nitrogen gas across of the knife holder, we found that RNAlater preserved tissues could be routinely sectioned with sufficient robustness at the desired thickness without crumpling or sticking to the cutting blade. The second issue with RNAlater-preserved tissues is that its components are inhibitive to proper immunolabelling (Brown and Smith 2009), probably as a consequence of their interference with the antigen-antibody interaction. Therefore, IF labelling with these sections could only be performed after RNAlater was completely replaced with an RVC-containing solution. With these easily adaptable modifications, we were able to obtain high quality transcriptomes from IF-guided microdissection of tissues preserved by RNAlater. Similar to the snap-frozen tissues, we used both anti-Lyve1 and anti-cytokeratin (PanCK) antibodies to demonstrate the validity of the modified immuno-LCM-RNAseq protocol. As shown in Supplementary Figure S11, in the presence of 10 mM RVC, high quality IF images were obtained with the mouse lacteal and stomach lymphatic vessels. With ∼1,500 cytokeratin positive (panCK) cells from the crypt region of the mouse small intestine, we obtained ∼15 million clean reads with an 82% overall mapping rate (Supplementary Table S2). About 14,000 expressed genes were detected at FPKM ≥1, similar to that with snap-frozen tissues (Supplementary Table S2). Furthermore, the transcriptomes from the two different preservation methods were also in high agreement with an average R value of 0.84 (Supplementary Figure S12, Supplementary Table S2).

As a further demonstration, we applied our approach to the analysis of an RNAlater preserved clinical sample, the human jejunum. Using the anti-Podoplanin antibody to identify lymphatic cells in these tissue sections, we micro-dissected ∼100 Podoplanin-positive cells to profile their gene expression (Figure 8). With 43 million clean reads, we were able to detect 15,000 genes expressed in these lymphatic endothelial cells (Supplementary Table S2, S7). To lend further validity of this result, we compared our transcriptome to a known expression profile of the human primary dermal lymphatic endothelial cells which is based on bulk RNAseq (Breschi et al., 2020). The resulting Pearson correlation between these two samples is 0.89 (R = 0.89), demonstrating the reliability of our method to characterize clinical samples.

## Discussion

Laser capture microdissection (LCM) (Emmert-Buck et al., 1996) offers a unique ability to precisely isolate targeted materials from either snap-frozen or RNAlater preserved bio-sections, even down to single cell levels. When combined with well-characterized phenotype markers, such as antibodies, it has long been known that LCM could provide detailed, spatially defined and cell-type guided transcriptome analysis with unparalleled precision. However, the key for the successful incorporation of this technology into the repertoire of spatial transcriptome methods is the protection of the RNA integrity during the lengthy process: the IF labelling often requires many hours to complete and the microdissection procedure could also take a long time to finish. In these processes, both endogenous and exogenous (such as airborne) RNases can quickly degrade the RNA exposed in the sectioned tissues. Although various schemes and protocols have been proposed to overcome this problem, the RNA quality issues remained unresolved until now. This is true even for formalin-fixed and paraffin-embedded (FFPE) tissue samples (Murray 2018; Foley et al., 2019). As demonstrated in this study, with the inclusion of a moderate amount of a small ribonuclease inhibitor, RVC, in most steps that must be performed under ambient conditions, RNA can be effectively protected with high quality RNA profiles acquired from as little as a few tens of dissected cells, mostly owing to their small size and fast diffusion in the dense tissue sections, which had not been explored before (de Heredia and Jansen 2004; Ohlson et al., 2005; Zielinski et al., 2006; Graindorge et al., 2019). It is as important that RVC has negligible effect on the actions of both primary and secondary antibodies and allows the immunolabelling to proceed according to the standard procedures. Our method also significantly out-performs results recently obtained using the Rapid protocol with bone marrow tissue (Baccin et al., 2020) in terms of number of genes as well as overall mapping rate, and rRNA, exonic, intronic, and intergenic mapping rates (Supplementary Figure S13, Supplementary Table S2). Given the simplicity of our approach, we anticipate that immuno-LCM-RNAseq can be adopted with most existing LCM platforms, facilitating its use in refined analysis of the spatial transcriptomes of intricately structured, phenotype complex tissues in their native state. As exemplified in the spatially select analysis of the lacteal capillary in the mouse small intestine, critical differences at the transcript isoform level could be resolved with a sensitivity unravelled by any other approach at this spatial scale.

Although with the present approach, as few as ∼60 cells were demonstrated to be sufficient to acquire high quality transcriptomes, there should still be room for further improvement. Indeed, LCM has the ability to isolate single cells, and even single nuclei (Guo et al., 2012). In our particular protocol, the RNA extraction process is associated with a certain amount of material loss, especially when the collected materials are extremely low. For example, for 100 cells, the total mRNA contained is probably less than half a nanogram. In this regard, adopting a single tube approach might further improve the lower limit of immuno-LCM-RNAseq substantially. Hence, based on what has been documented in the literature (Tang et al., 2009; Soumillon et al., 2014), analysis at the single cell level might be feasible. However, when pushed to such a limit, whether it is still possible to achieve full-length transcript and isoform analysis remains to be established.

It should also be noted that with either snap-frozen or RNAlater preservation, the tissues should still retain their native state if the protocols are performed properly. Therefore, immuno-LCM-RNAseq allows for the native transcriptome, i.e., under *in vivo* conditions and environments, to be acquired. In comparison to the “standard” transcriptomes derived from scRNAseq profiles, the transcriptomes acquired with immuno-LCM-RNAseq are not only more comprehensive, but even more importantly, obtained under exactly the same conditions. In fact, potential artefacts owing to single cell preparation procedures notwithstanding (van den Brink et al., 2017), even the best separated clusters in a single cell dataset cannot provide a detailed profile with isoforms quantitatively resolved as in the immuno-LCM-RNAseq transcriptomes.

In summary, we have established a powerful method for acquiring spatial transcriptional programs based on immuno-guided LCM with exceptional RNA quality to allow full length transcript analysis. With this unprecedented capability, rare cell types in particular or cells whose functioning is exquisitely sensitive to their positioning or local environment within the tissue can now be interrogated to obtain an understanding of their complete transcriptome, including transcript isoforms from which both cell intrinsic and cell-cell signalling networks may be delineated. It is only with such a systems-wide characterization of the cells within the native tissue can their inter-related functioning be fully understood, which is ultimately essential for an understanding of the functioning of the tissues as a whole, whether healthy or diseased.

## Data Availability

The datasets presented in this study can be found in online repositories. The names of the repository/repositories and accession number(s) can be found below: https://www.ncbi.nlm.nih.gov/bioproject?term=PRJNA658865.

## References

[B1] Abella J. V. G. Galloni C. Pernier J. Barry D. J. Kjær S. Carlier M.-F. (2015). Isoform Diversity in the Arp2/3 Complex Determines Actin Filament Dynamics. Nat. Cel Biol. 18, 76–86. 10.1038/ncb3286 26655834

[B2] Agarwal N. Adhikari A. S. Iyer S. V. Hekmatdoost K. Welch D. R. Iwakuma T. (2013). MTBP Suppresses Cell Migration and Filopodia Formation by Inhibiting ACTN4. Oncogene 32, 462–470. 10.1038/onc.2012.69 22370640 PMC3742333

[B3] Alon S. Goodwin D. R. Sinha A. Wassie A. T. Chen F. Daugharthy E. R. (2021). Expansion Sequencing: Spatially Precise *In Situ* Transcriptomics in Intact Biological Systems. Science 371, eaax2656. 10.1126/science.aax2656 33509999 PMC7900882

[B4] Andrews S. (2010). FastQC: A Quality Control Tool for High Throughput Sequence Data. Available at: https://www.bioinformatics.babraham.ac.uk/projects/fastqc/ .

[B5] Arizti-Sanz J. Freije C. A. Stanton A. C. Petros B. A. Boehm C. K. Siddiqui S. (2020). Streamlined Inactivation, Amplification, and Cas13-Based Detection of SARS-CoV-2. Nat. Commun. 11, 5921. 10.1038/s41467-020-19097-x 33219225 PMC7680145

[B6] Asp M. Bergenstråhle J. Lundeberg J. (2020). Spatially Resolved Transcriptomes-Next Generation Tools for Tissue Exploration. BioEssays 42, 1900221. 10.1002/bies.201900221 32363691

[B7] Baccin C. Al-Sabah J. Velten L. Helbling P. M. Grünschläger F. Hernández-Malmierca P. (2020). Combined Single-Cell and Spatial Transcriptomics Reveal the Molecular, Cellular and Spatial Bone Marrow Niche Organization. Nat. Cel Biol. 22, 38–48. 10.1038/s41556-019-0439-6 PMC7610809 31871321

[B8] Bath C. Fink T. Vorum H. Hjortdal J. Zachar V. (2014). Technical Brief: Optimized Pipeline for Isolation of High-Quality RNA from Corneal Cell Subpopulations. Mol. Vis. 20, 797–803. 24940035 PMC4057246

[B9] Berger S. L. Birkenmeier C. S. (1979). Inhibition of Intractable Nucleases with Ribonucleoside Vanadyl Complexes: Isolation of Messenger Ribonucleic Acid from Resting Lymphocytes. Biochemistry 18, 5143–5149. 10.1021/bi00590a018 497174

[B10] Bernier-Latmani J. Petrova T. V. (2017). Intestinal Lymphatic Vasculature: Structure, Mechanisms and Functions. Nat. Rev. Gastroenterol. Hepatol. 14, 510–526. 10.1038/nrgastro.2017.79 28655884

[B11] Bolger A. M. Lohse M. Usadel B. (2014). Trimmomatic: a Flexible Trimmer for Illumina Sequence Data. Bioinformatics 30, 2114–2120. 10.1093/bioinformatics/btu170 24695404 PMC4103590

[B12] Brady G. Boggan L. Bowie A. O'Neill L. A. J. (2005). Schlafen-1 Causes a Cell Cycle Arrest by Inhibiting Induction of Cyclin D1. J. Biol. Chem. 280, 30723–30734. 10.1074/jbc.M500435200 15946944

[B13] Breschi A. Muñoz-Aguirre M. Wucher V. Davis C. A. Garrido-Martín D. Djebali S. (2020). A Limited Set of Transcriptional Programs Define Major Cell Types. Genome Res. 30, 1047–1059. 10.1101/gr.263186.120 32759341 PMC7397875

[B14] Brown A. L. Smith D. W. (2009). Improved RNA Preservation for Immunolabeling and Laser Microdissection. RNA 15, 2364–2374. 10.1261/rna.1733509 19850907 PMC2779672

[B15] Clément-Ziza M. Munnich A. Lyonnet S. Jaubert F. Besmond C. (2008). Stabilization of RNA during Laser Capture Microdissection by Performing Experiments under Argon Atmosphere or Using Ethanol as a Solvent in Staining Solutions. RNA 14, 2698–2704. 10.1261/rna.1261708 18945804 PMC2590969

[B16] Credle J. J. Itoh C. Y. Yuan T. Sharma R. Scott E. R. Workman R. E. (2017). Multiplexed Analysis of Fixed Tissue RNA Using Ligation *In Situ* Hybridization. Nucleic Acids Res. 45, e128. 10.1093/nar/gkx471 28854731 PMC5737328

[B17] Crosetto N. Bienko M. van Oudenaarden A. (2015). Spatially Resolved Transcriptomics and beyond. Nat. Rev. Genet. 16, 57–66. 10.1038/nrg3832 25446315

[B18] Davies C. d. L. Berk D. A. Pluen A. Jain R. K. (2002). Comparison of IgG Diffusion and Extracellular Matrix Composition in Rhabdomyosarcomas Grown in Mice versus *In Vitro* as Spheroids Reveals the Role of Host Stromal Cells. Br. J. Cancer 86, 1639–1644. 10.1038/sj.bjc.6600270 12085216 PMC2746604

[B19] Dey P. (2018). “Frozen Section: Principle and Procedure,” in Basic and Advanced Laboratory Techniques in Histopathology and Cytology (Singapore: Springer), 51–55. 10.1007/978-981-10-8252-8_6

[B20] Di Paolo A. Garat J. Eastman G. Farias J. Dajas-Bailador F. Smircich P. (2021). Functional Genomics of Axons and Synapses to Understand Neurodegenerative Diseases. Front. Cel. Neurosci. 15, 686722. 10.3389/fncel.2021.686722 PMC8267896 34248504

[B21] Diaz Z. Aguilar-Mahecha A. Paquet E. R. Basik M. Orain M. Camlioglu E. (2013). Next-generation Biobanking of Metastases to Enable Multidimensional Molecular Profiling in Personalized Medicine. Mod. Pathol. 26, 1413–1424. 10.1038/modpathol.2013.81 23743930

[B22] Dickson K. A. Haigis M. C. Raines R. T. (2005). Ribonuclease Inhibitor: Structure and Function. Prog. Nucleic Acid Res. Mol. Biol. 80, 349–374. 10.1016/S0079-6603(05)80009-1 16164979 PMC2811166

[B23] Ellis M. Davis N. Coop A. Liu M. Schumaker L. Lee R. Y. (2002). Development and Validation of a Method for Using Breast Core Needle Biopsies for Gene Expression Microarray Analyses. Clin. Cancer Res. 8, 1155–1166. 12006532

[B24] Emmert-Buck M. R. Bonner R. F. Smith P. D. Chuaqui R. F. Zhuang Z. Goldstein S. R. (1996). Laser Capture Microdissection. Science 274, 998–1001. 10.1126/science.274.5289.998 8875945

[B25] Fend F. Emmert-Buck M. R. Chuaqui R. Cole K. Lee J. Liotta L. A. (1999). Immuno-LCM: Laser Capture Microdissection of Immunostained Frozen Sections for mRNA Analysis. Am. J. Pathol. 154, 61–66. 10.1016/S0002-9440(10)65251-0 9916919 PMC1853427

[B26] Florell S. R. Coffin C. M. Holden J. A. Zimmermann J. W. Gerwels J. W. Summers B. K. (2001). Preservation of RNA for Functional Genomic Studies: A Multidisciplinary Tumor Bank Protocol. Mod. Pathol. 14, 116–128. 10.1038/modpathol.3880267 11235903

[B27] Foley J. W. Zhu C. Jolivet P. Zhu S. X. Lu P. Meaney M. J. (2019). Gene Expression Profiling of Single Cells from Archival Tissue with Laser-Capture Microdissection and Smart-3SEQ. Genome Res. 29, 1816–1825. 10.1101/gr.234807.118 31519740 PMC6836736

[B28] Gallego Romero I. Pai A. A. Tung J. Gilad Y. (2014). RNA-seq: Impact of RNA Degradation on Transcript Quantification. BMC Biol. 12, 42. 10.1186/1741-7007-12-42 24885439 PMC4071332

[B29] Ge S. X. Jung D. Yao R. (2020). ShinyGO: a Graphical Gene-Set Enrichment Tool for Animals and Plants. Bioinformatics 36, 2628–2629. 10.1093/bioinformatics/btz931 31882993 PMC7178415

[B30] Graindorge A. Pinheiro I. Nawrocka A. Mallory A. C. Tsvetkov P. Gil N. (2019). In-cell Identification and Measurement of RNA-Protein Interactions. Nat. Commun. 10, 5317. 10.1038/s41467-019-13235-w 31757954 PMC6876571

[B31] Grgic I. Kaistha B. P. Hoyer J. Köhler R. (2009). Endothelial Ca2+-Activated K+ Channels in normal and Impaired EDHF-Dilator Responses - Relevance to Cardiovascular Pathologies and Drug Discovery. Br. J. Pharmacol. 157, 509–526. 10.1111/j.1476-5381.2009.00132.x 19302590 PMC2707963

[B32] Grimm J. Mueller A. Hefti F. Rosenthal A. (2004). Molecular Basis for Catecholaminergic Neuron Diversity. Proc. Natl. Acad. Sci. 101, 13891–13896. 10.1073/pnas.0405340101 15353588 PMC518849

[B33] Guo Y. Yang Y. Zhou J. Czajkowsky D. M. Liu B. Shao Z. (2012). Microdissection of Spatially Identified Single Nuclei in a Solid Tumor for Single Cell Whole Genome Sequencing. Biotechniques 52, 1–3. 10.2144/000113860 26307250

[B34] Hanahan D. Weinberg R. A. (2011). Hallmarks of Cancer: The Next Generation. Cell 144, 646–674. 10.1016/j.cell.2011.02.013 21376230

[B35] Hawrylycz M. J. Lein E. S. Guillozet-Bongaarts A. L. Shen E. H. Ng L. Miller J. A. (2012). An Anatomically Comprehensive Atlas of the Adult Human Brain Transcriptome. Nature 489, 391–399. 10.1038/nature11405 22996553 PMC4243026

[B36] He Q. Shi J. Sun H. An J. Huang Y. Sheikh M. S. (2010). Characterization of Human Homeodomain-Interacting Protein Kinase 4 (HIPK4) as a Unique Member of the HIPK Family. Mol. Cel. Pharmacol. 2, 61–68. 10.4255/mcpharmacol.10.09 PMC2876313 20508833

[B37] Hentze J. L. Kringelbach T. M. Novotny G. W. Hamid B. H. Ravn V. Christensen I. J. (2019). Optimized Biobanking Procedures for Preservation of RNA in Tissue: Comparison of Snap-Freezing and RNAlater-Fixation Methods. Biopreserv. Biobank. 17, 562–569. 10.1089/bio.2019.0028 31618057

[B38] Hofsteenge J. (1997). “Ribonuclease Inhibitor,” in Ribonucleases Structures and Functions. Editors D'Alessio G. Riordan J. F. (New York: Academic Press), 621–658. 10.1016/b978-012588945-2/50020-0

[B39] Im K. Mareninov S. Diaz M. F. P. Yong W. H. (2019). An Introduction to Performing Immunofluorescence Staining. Methods Mol. Biol., 1897, 299–311. 10.1007/978-1-4939-8935-5_26 30539454 PMC6918834

[B40] Kamakura S. Moriguchi T. Nishida E. (1999). Activation of the Protein Kinase ERK5/BMK1 by Receptor Tyrosine Kinases. J. Biol. Chem. 274, 26563–26571. 10.1074/jbc.274.37.26563 10473620

[B41] Kasahara T. Miyazaki T. Nitta H. Ono A. Miyagishima T. Nagao T. (2006). Evaluation of Methods for Duration of Preservation of RNA Quality in Rat Liver Used for Transcriptome Analysis. J. Toxicol. Sci. 31, 509–519. 10.2131/jts.31.509 17202763

[B42] Kim D. Langmead B. Salzberg S. L. (2015). HISAT: a Fast Spliced Aligner with Low Memory Requirements. Nat. Methods 12, 357–360. 10.1038/nmeth.3317 25751142 PMC4655817

[B43] Kopylova E. Noé L. Touzet H. (2012). SortMeRNA: Fast and Accurate Filtering of Ribosomal RNAs in Metatranscriptomic Data. Bioinformatics 28, 3211–3217. 10.1093/bioinformatics/bts611 23071270

[B44] Kuang C.-y. Yang T.-h. Zhang Y. Zhang L. Wu Q. (2014). Schlafen 1 Inhibits the Proliferation and Tube Formation of Endothelial Progenitor Cells. PLoS One 9, e109711. 10.1371/journal.pone.0109711 25329797 PMC4199616

[B45] Lau J. Y. N. Qian K.-P. Wu P. C. Davis G. L. (1993). Ribonucleotide Vanadyl Complexes Inhibit Polymerase Chain Reaction. Nucl. Acids Res. 21, 2777. 10.1093/nar/21.11.2777 8392707 PMC309629

[B46] Legres L. G. Janin A. Masselon C. Bertheau P. (2014). Beyond Laser Microdissection Technology: Follow the Yellow brick Road for Cancer Research. Am. J. Cancer Res. 4, 1–28. 24482735 PMC3902229

[B47] Leon-Lai C. H. Gresser M. J. Tracey A. S. (1996). Influence of Vanadium(V) Complexes on the Catalytic Activity of Ribonuclease A. The Role of Vanadate Complexes as Transition State Analogues to Reactions at Phosphate. Can. J. Chem. 74, 38–48. 10.1139/v96-005

[B48] Liao J. Lu X. Shao X. Zhu L. Fan X. (2021). Uncovering an Organ's Molecular Architecture at Single-Cell Resolution by Spatially Resolved Transcriptomics. Trends Biotechnol. 39, 43–58. 10.1016/j.tibtech.2020.05.006 32505359

[B49] Lienhard G. E. Secemski I. I. Koehler K. A. Lindquist R. N. (1972). Enzymatic Catalysis and the Transition State Theory of Reaction Rates: Transition State Analogs. Cold Spring Harb Symp Quant Biol. 36, 45–51. 10.1101/sqb.1972.036.01.009 4508159

[B50] Lopez de Heredia M. Jansen R. P. (2004). RNA Integrity as a Quality Indicator during the First Steps of RNP Purifications : A Comparison of Yeast Lysis Methods. BMC Biochem. 5, 14. 10.1186/1471-2091-5-14 15461782 PMC524479

[B51] Love M. I. Huber W. Anders S. (2014). Moderated Estimation of Fold Change and Dispersion for RNA-Seq Data with DESeq2. Genome Biol. 15, 550. 10.1186/s13059-014-0550-8 25516281 PMC4302049

[B52] Lyubimova A. Itzkovitz S. Junker J. P. Fan Z. P. Wu X. van Oudenaarden A. (2013). Single-molecule mRNA Detection and Counting in Mammalian Tissue. Nat. Protoc. 8, 1743–1758. 10.1038/nprot.2013.109 23949380

[B53] Martin M. (2011). Cutadapt Removes Adapter Sequences from High-Throughput Sequencing Reads. EMBnet j. 17, 10. 10.14806/ej.17.1.200

[B54] Marx V. (2021). Method of the Year: Spatially Resolved Transcriptomics. Nat. Methods 18, 9–14. 10.1038/s41592-020-01033-y 33408395

[B55] Mita Y. Uchida R. Yasuhara S. Kishi K. Hoshi T. Matsuo Y. (2021). Identification of a Novel Endogenous Long Non-coding RNA that Inhibits Selenoprotein P Translation. Nucleic Acids Res. 49, 6893–6907. 10.1093/nar/gkab498 34142161 PMC8266573

[B56] Moor A. E. Itzkovitz S. (2017). Spatial Transcriptomics: Paving the Way for Tissue-Level Systems Biology. Curr. Opin. Biotechnol. 46, 126–133. 10.1016/j.copbio.2017.02.004 28346891

[B57] Murakami H. Liotta L. Star R. A. (2000). IF-LCM: Laser Capture Microdissection of Immunofluorescently Defined Cells for mRNA Analysis. Kidney Int. 58, 1346–1353. 10.1046/j.1523-1755.2000.00295.x 10972700

[B58] Murray G. I. (2018). Laser Capture Microdissection Methods and Protocols. 3nd ed. New York: Humana Press.

[B59] Mutter G. L. Zahrieh D. Liu C. Neuberg D. Finkelstein D. Baker H. E. (2004). Comparison of Frozen and RNALater Solid Tissue Storage Methods for Use in RNA Expression Microarrays. BMC Genomics 5, 88. 10.1186/1471-2164-5-88 15537428 PMC534099

[B60] Nguyen A. Khoo W. H. Moran I. Croucher P. I. Phan T. G. (2018). Single Cell RNA Sequencing of Rare Immune Cell Populations. Front. Immunol. 9, 1553. 10.3389/fimmu.2018.01553 30022984 PMC6039576

[B61] Nichterwitz S. Chen G. Aguila Benitez J. Yilmaz M. Storvall H. Cao M. (2016). Laser Capture Microscopy Coupled with Smart-Seq2 for Precise Spatial Transcriptomic Profiling. Nat. Commun. 7, 12139. 10.1038/ncomms12139 27387371 PMC4941116

[B62] Ohlson J. Ensterö M. Sjöberg B. M. Ohman M. (2005). A Method to Find Tissue-specific Novel Sites of Selective Adenosine Deamination. Nucleic Acids Res. 33, e167. 10.1093/nar/gni169 16257978 PMC1275595

[B63] Páska C. Bögi K. Szilák L. Tőkés A. Szabó E. Sziller I. (2004). Effect of Formalin, Acetone, and RNAlater Fixatives on Tissue Preservation and Different Size Amplicons by Real-Time PCR from Paraffin-Embedded Tissue. Diagn. Mol. Pathol. 13, 234–240. 10.1097/01.pdm.0000134778.37729.9f 15538114

[B64] Patel H. P. Brouwer I. Lenstra T. L. (2021). Optimized Protocol for Single-Molecule RNA FISH to Visualize Gene Expression in *S. cerevisiae* . STAR Protoc. 2, 100647. 10.1016/j.xpro.2021.100647 34278333 PMC8264745

[B65] Pearson G. Robinson F. Beers Gibson T. Xu B.-e. Karandikar M. Berman K. (2001). Mitogen-Activated Protein (MAP) Kinase Pathways: Regulation and Physiological Functions*. Endocr. Rev. 22, 153–183. 10.1210/edrv.22.2.0428 11294822

[B66] Peng G. Suo S. Chen J. Chen W. Liu C. Yu F. (2016). Spatial Transcriptome for the Molecular Annotation of Lineage Fates and Cell Identity in Mid-gastrula Mouse Embryo. Developmental Cel 36, 681–697. 10.1016/j.devcel.2016.02.020 27003939

[B67] Peng G. Suo S. Cui G. Yu F. Wang R. Chen J. (2019). Molecular Architecture of Lineage Allocation and Tissue Organization in Early Mouse Embryo. Nature 572, 528–532. 10.1038/s41586-019-1469-8 31391582

[B68] Pertea M. Kim D. Pertea G. M. Leek J. T. Salzberg S. L. (2016). Transcript-level Expression Analysis of RNA-Seq Experiments with HISAT, StringTie and Ballgown. Nat. Protoc. 11, 1650–1667. 10.1038/nprot.2016.095 27560171 PMC5032908

[B69] Pertea M. Pertea G. M. Antonescu C. M. Chang T.-C. Mendell J. T. Salzberg S. L. (2015). StringTie Enables Improved Reconstruction of a Transcriptome from RNA-Seq Reads. Nat. Biotechnol. 33, 290–295. 10.1038/nbt.3122 25690850 PMC4643835

[B70] Popella L. Jung J. Popova K. Ðurica-Mitić S. Barquist L. Vogel J. (2021). Global RNA Profiles Show Target Selectivity and Physiological Effects of Peptide-Delivered Antisense Antibiotics. Nucleic Acids Res. 49, 4705–4724. 10.1093/nar/gkab242 33849070 PMC8096218

[B71] Qian J. Boswell S. A. Chidley C. Lu Z.-x. Pettit M. E. Gaudio B. L. (2020). An Enhanced Isothermal Amplification Assay for Viral Detection. Nat. Commun. 11, 5920. 10.1038/s41467-020-19258-y 33219228 PMC7679446

[B72] Qin T.-T. Xu G.-C. Qi J.-W. Yang G.-L. Zhang K. Liu H.-L. (2015). Tumour Necrosis Factor Superfamily Member 15 (Tnfsf15) Facilitates Lymphangiogenesis via Up-Regulation of Vegfr3 Gene Expression in Lymphatic Endothelial Cells. J. Pathol. 237, 307–318. 10.1002/path.4577 26096340

[B73] Rao A. Barkley D. França G. S. Yanai I. (2021). Exploring Tissue Architecture Using Spatial Transcriptomics. Nature 596, 211–220. 10.1038/s41586-021-03634-9 34381231 PMC8475179

[B74] Rodriques S. G. Stickels R. R. Goeva A. Martin C. A. Murray E. Vanderburg C. R. (2019). Slide-seq: A Scalable Technology for Measuring Genome-wide Expression at High Spatial Resolution. Science 363, 1463–1467. 10.1126/science.aaw1219 30923225 PMC6927209

[B75] Russo A. Acharya K. R. Shapiro R. (2001). Small Molecule Inhibitors of RNase A and Related Enzymes. Meth. Enzymol. 341, 629–648. 10.1016/s0076-6879(01)41181-5 11582810

[B76] Shapiro R. (2001). Cytoplasmic Ribonuclease Inhibitor. Meth. Enzymol. 341, 611–628. 10.1016/s0076-6879(01)41180-3 11582809

[B77] Shieh T.-M. Chen C.-Y. Hsueh C. Yu C.-C. Chen C.-C. Wang T.-H. (2018). Application of Ribonucleoside Vanadyl Complex (RVC) for Developing a Multifunctional Tissue Preservative Solution. PLoS One 13, e0194393. 10.1371/journal.pone.0194393 29538436 PMC5851642

[B78] Soumillon M. Cacchiarelli D. Semrau S. van Oudenaarden A. Mikkelsen T. S. (2014). Characterization of Directed Differentiation by High-Throughput Single-Cell RNA-Seq. bioRxiv. 10.1101/003236

[B79] Tang F. Barbacioru C. Wang Y. Nordman E. Lee C. Xu N. (2009). mRNA-Seq Whole-Transcriptome Analysis of a Single Cell. Nat. Methods 6, 377–382. 10.1038/nmeth.1315 19349980

[B80] Tangrea M. A. Hanson J. C. Bonner R. F. Pohida T. J. Rodriguez-Canales J. Emmert-Buck M. R. (2011b). Immunoguided Microdissection Techniques. Methods Mol. Biol. 755, 57–66. 10.1007/978-1-61779-163-5_4 21761293 PMC4794997

[B81] Tangrea M. A. Mukherjee S. Gao B. Markey S. P. Du Q. Armani M. (2011a). Effect of Immunohistochemistry on Molecular Analysis of Tissue Samples. J. Histochem. Cytochem. 59, 591–600. 10.1369/0022155411404704 21430260 PMC3201191

[B82] Uhlén M. Fagerberg L. Hallström B. M. Lindskog C. Oksvold P. Mardinoglu A. (2015). Tissue-based Map of the Human Proteome. Science 347, 1260419. 10.1126/science.1260419 25613900

[B83] van den Brink S. C. Sage F. Vértesy Á. Spanjaard B. Peterson-Maduro J. Baron C. S. (2017). Single-cell Sequencing Reveals Dissociation-Induced Gene Expression in Tissue Subpopulations. Nat. Methods 14, 935–936. 10.1038/nmeth.4437 28960196

[B84] Vickovic S. Eraslan G. Salmén F. Klughammer J. Stenbeck L. Schapiro D. (2019). High-definition Spatial Transcriptomics for *In Situ* Tissue Profiling. Nat. Methods 16, 987–990. 10.1038/s41592-019-0548-y 31501547 PMC6765407

[B85] Wacker S. A. Alvarado C. von Wichert G. Knippschild U. Wiedenmann J. Clauss K. (2010). RITA, a Novel Modulator of Notch Signalling, Acts via Nuclear export of RBP-J-J. EMBO J. 30, 43–56. 10.1038/emboj.2010.289 21102556 PMC3020113

[B86] Walker D. G. Whetzel A. M. Serrano G. Sue L. I. Lue L.-F. Beach T. G. (2016). Characterization of RNA Isolated from Eighteen Different Human Tissues: Results from a Rapid Human Autopsy Program. Cell Tissue Bank 17, 361–375. 10.1007/s10561-016-9555-8 27083469 PMC5026224

[B87] Wang L. Nie J. Sicotte H. Li Y. Eckel-Passow J. E. Dasari S. (2016). Measure Transcript Integrity Using RNA-Seq Data. BMC Bioinformatics 17, 58. 10.1186/s12859-016-0922-z 26842848 PMC4739097

[B88] Wang L. Wang S. Li W. (2012). RSeQC: Quality Control of RNA-Seq Experiments. Bioinformatics 28, 2184–2185. 10.1093/bioinformatics/bts356 22743226

[B89] Wei A. D. Gutman G. A. Aldrich R. Chandy K. G. Grissmer S. Wulff H. (2005). International Union of Pharmacology. LII. Nomenclature and Molecular Relationships of Calcium-Activated Potassium Channels. Pharmacol. Rev. 57, 463–472. 10.1124/pr.57.4.9 16382103

[B90] Zielinski J. Kilk K. Peritz T. Kannanayakal T. Miyashiro K. Y. Eiríksdóttir E. (2006). *In Vivo* identification of Ribonucleoprotein-RNA Interactions. Proc. Natl. Acad. Sci. 103, 1557–1562. 10.1073/pnas.0510611103 16432185 PMC1345716

