# Supplementary material for: Robust Acquisition of Spatial Transcriptional Programs in Tissues With Immunofluorescence-Guided Laser Capture Microdissection
Source: Front Cell Dev Biol. 2022 Mar 25;10:853188. doi: 10.3389/fcell.2022.853188 (PMC8990165; doi:10.3389/fcell.2022.853188)
Supplement: Supplementary file 3 [file DataSheet1.PDF]

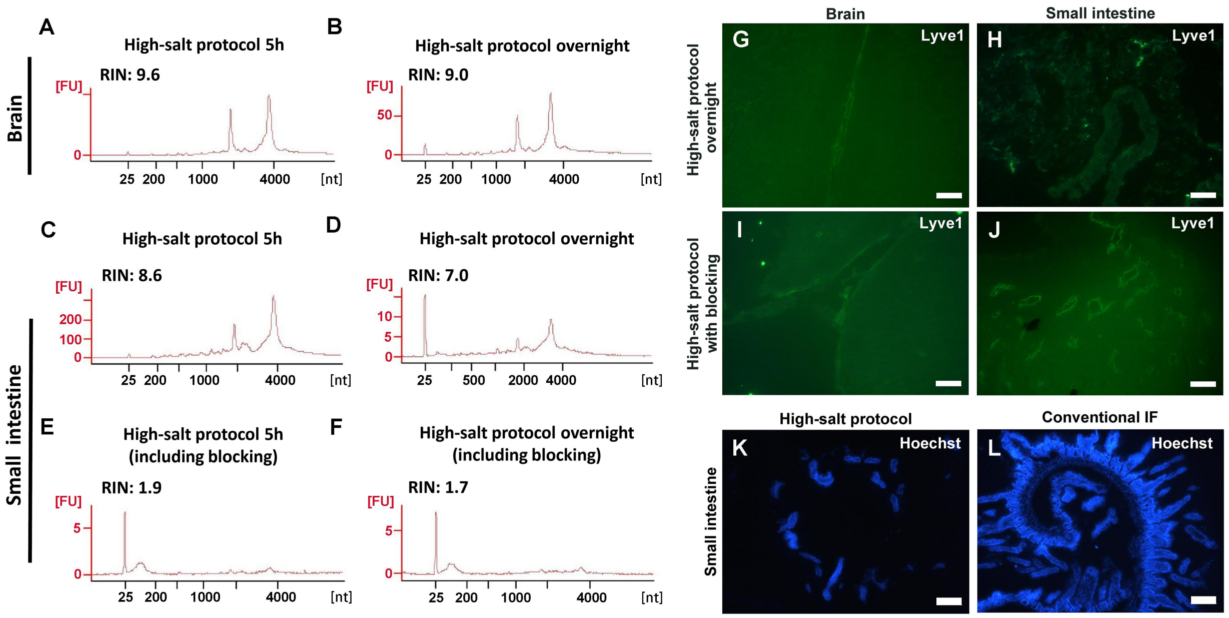

Figure S1: Effectiveness of the high-salt protocol to preserve RNA and produce high quality IF images of frozen tissue sections. (**A-D**) RNA quality of the mouse brain (**A, B**) and small intestine (**C, D**) frozen sections after a 5 hours (**A, C**) or overnight (**B, D**) high-salt (2 M NaCl) protocol. The original protocol described an overnight IF staining. This shorter, 5 hours-incubation was also examined here owing to the poor quality of the IF images on small intestine sections with the overnight incubation (**H**). (**E, F**) RNA quality of mouse small intestine frozen sections after the 5 hours (**E**) or overnight (**F**) high-salt protocol, including a blocking step in the staining procedure, showing degraded RNA. The original protocol did not include a blocking step, which might have contributed to the poor quality of IF images, and so we also examined this high-salt protocol with a blocking step. (**G, H**) IF images of the labelled lymphatic vessel cells in mouse brain (**G**) and small intestine (**H**) frozen sections following the overnight high-salt protocol, showing a lower signal-to-noise of the labeled cells than in the control (**Figure 2G-H**). (**I, J**) High-salt protocol labelled lymphatic vessel cells in the mouse brain (**I**) and small intestine (**J**) frozen sections, including a blocking step (~ 5 hours for the total procedure). (**K, L**) Images of the whole mouse small intestine tissue section after ~ 5 hours high-salt (**K**) or conventional IF (**L**), labelled by Hoechst. In the small intestine tissue sections, the high-salt protocol damaged the tissue section structure thus preventing further analysis. Scale bar: 50 µm (**G-J**), 200 µm (**K, L**).

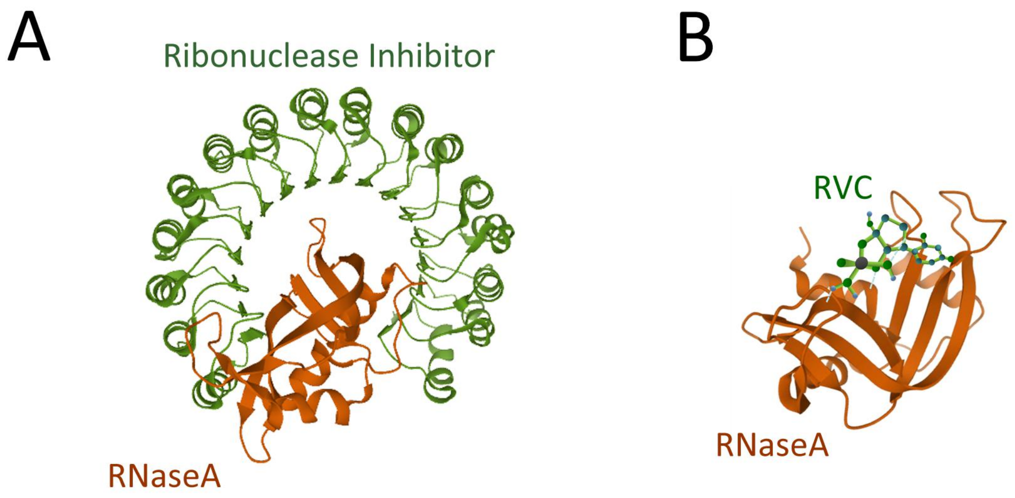

Figure S2: Structural models depicting the interaction of RNaseA with inhibitors. (**A**) The RNase inhibitor bound to RNaseA (pdb: 3TSR). (**B**) Model of RVC interacting within the active site of RNaseA to give a sense of the relative differences between the RNase inhibitor and RVC. This model was generated by manually positioning a typical component of RVC (the uridine vanadyl complex) within the active site of RNaseA.

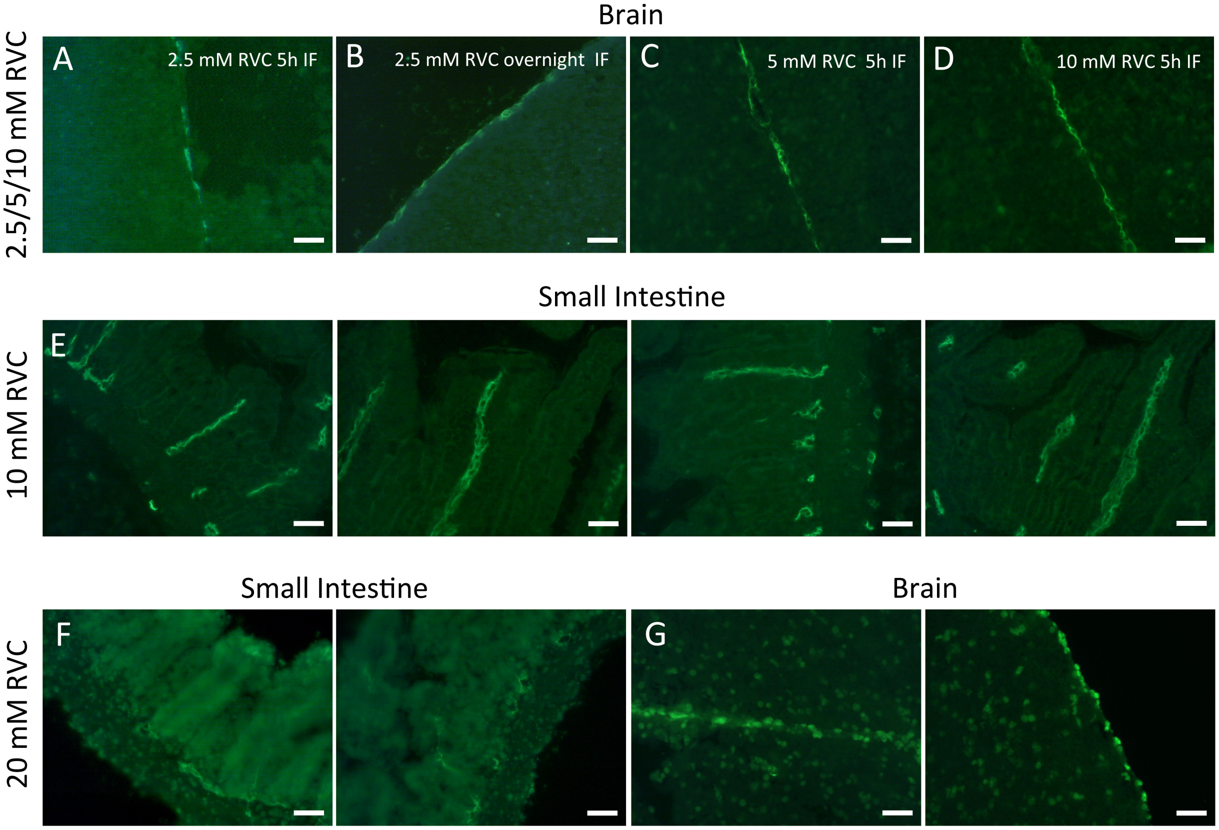

Figure S3: Quality of IF images following IF in the presence of RVC with different frozen tissue sections. (**A, B**) Images of mouse brain sections following a 5 hours (**A**) or overnight (**B**) IF staining in the presence of 2.5 mM RVC. Note that the image quality of 5 hours IF staining is as good as overnight incubation. (**C, D**) Images of mouse brain sections using 5 mM (**C**) or 10 mM (**D**) RVC during IF, showing that there is no negative effect of RVC on image quality (compare with **Figure 2H**). (**E**) Several examples of IF images of mouse small intestine sections following the RVC-based IF procedure (in 10 mM RVC). (**F, G**) Typical IF images obtained of small intestine (**F**) or brain (**G**) sections in the presence of 20 mM RVC following the RVC-based IF procedure. Note that there is higher background and less distinct labeling in these images compared to the 10 mM RVC-based IF procedure shown in (**E**). All samples were stained with anti-Lyve1 antibody. Scale bar: 50 µm.

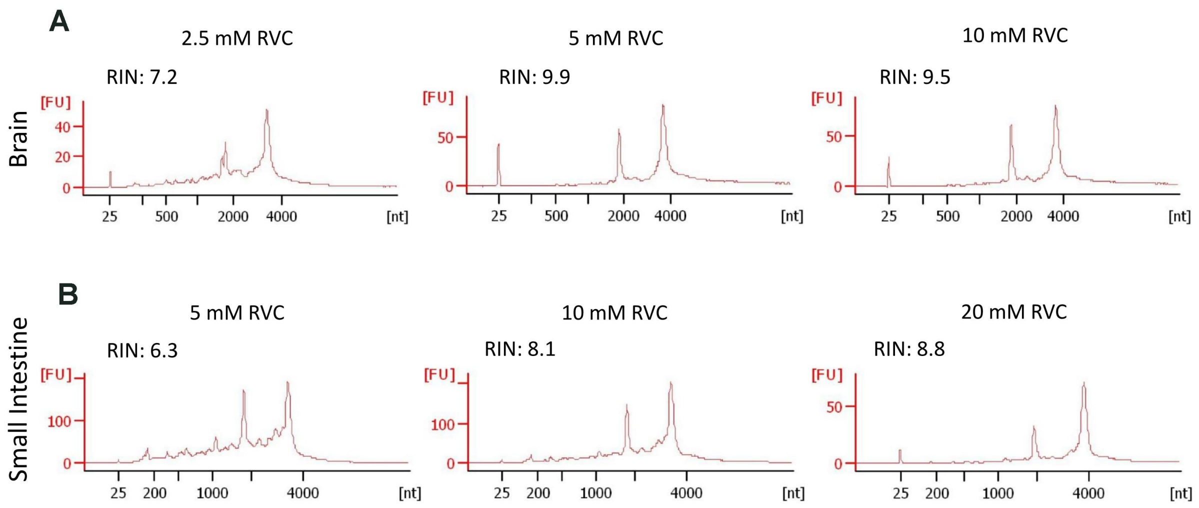

Figure S4: Effectiveness of RVC to maintain RNA quality during IF staining of mouse brain and small intestine frozen tissue sections. (**A, B**) The RNA quality of mouse brain (**A**) and small intestine frozen sections (**B**) in the presence of the indicated concentrations of RVC following IF (see Online Method). For brain tissues with low endogenous RNase, 5 mM RVC is sufficient to protect the RNA during the ~5 hours immunolabelling procedure, while for small intestine sections that have moderate levels of RNase, 10 mM RVC is needed to sufficiently preserve the RNA.

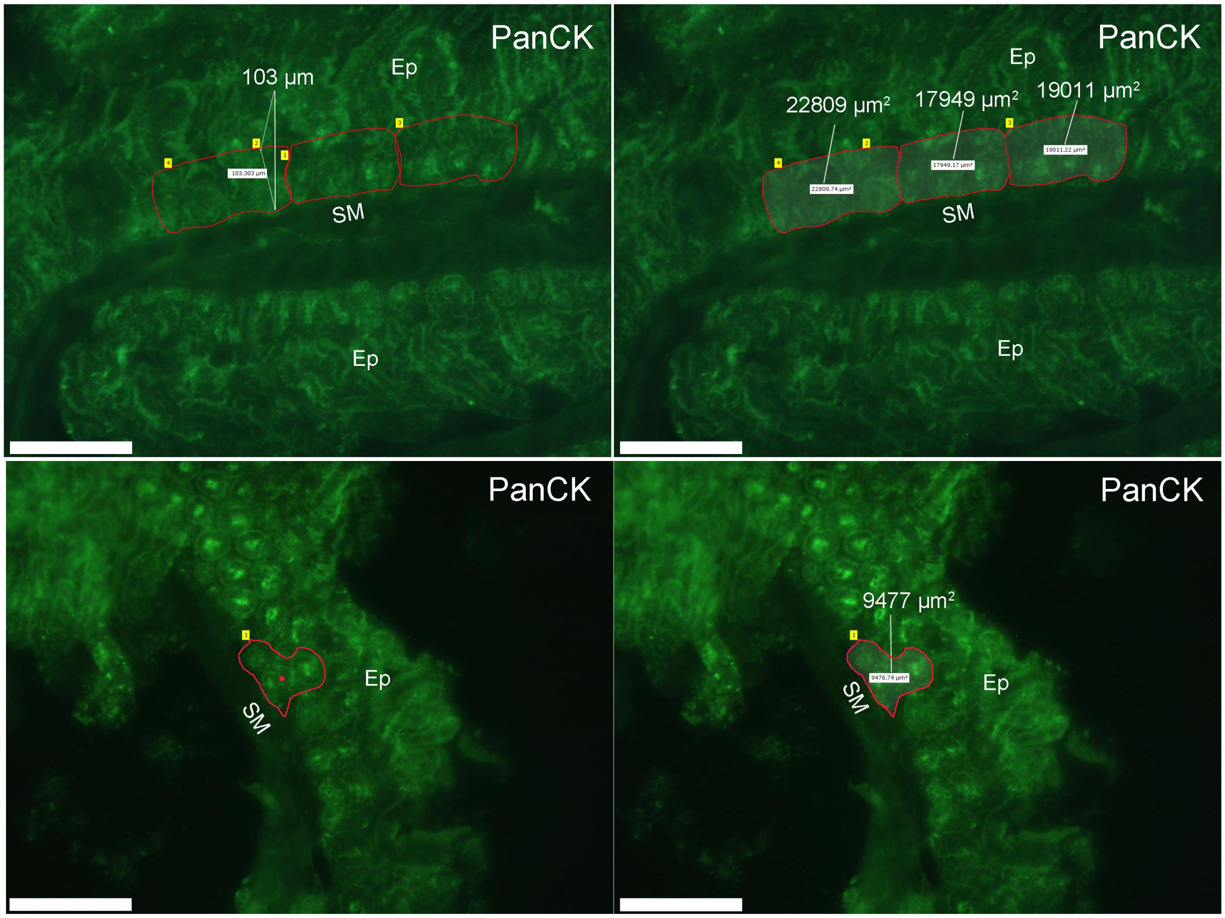

Figure S5: IF images of mouse small intestine epithelial cells used to calculate the cell numbers of laser-dissected samples. Examples of the IF images of PanCK-labelled epithelial cells (Ep) that were used to determine number of collected cells (see Supplementary Table 1). Ep cells only within ~100 µm of the submucosal layer (SM) were collected. Scale bar: 200 µm.

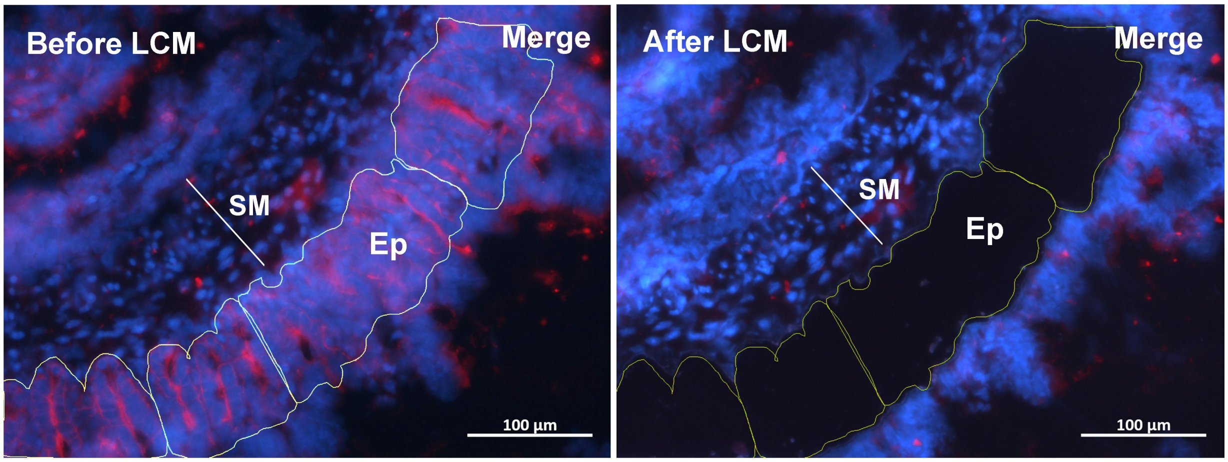

Figure S6: Laser microdissection of the cytokeratin positive epithelial cells (Ep) in the mouse small intestine tissue section. This image is of the same region as shown in **Figure 5** but here showing the nuclei of all cells (Red: PanCK, Blue: Hoechst). SM, submucosal layer; Ep, epithelial cells.

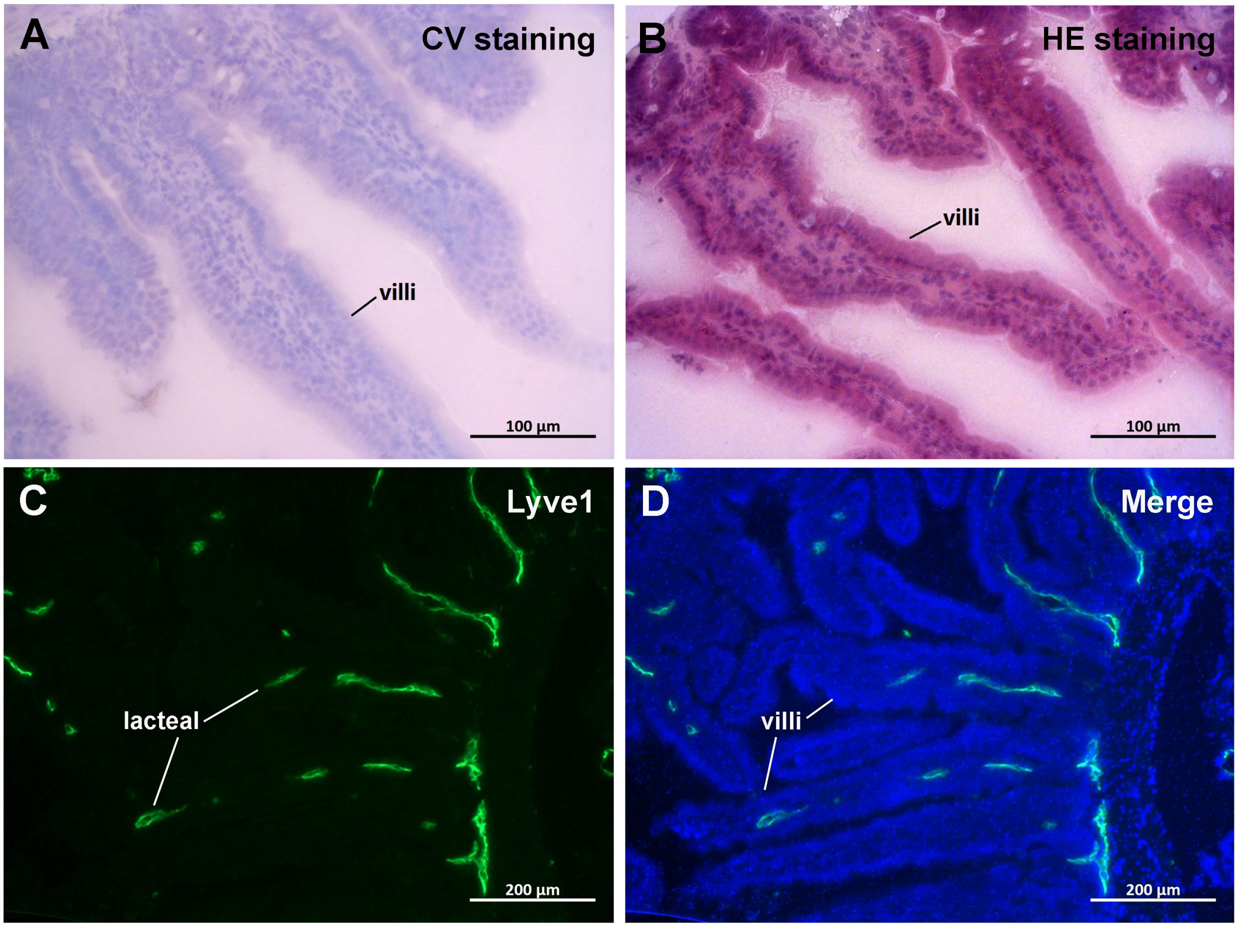

Figure S7: Histological staining and immunolabelling images of mouse small intestine villi and lacteal. (**A, B**) Cresyl violet and H&E staining of mouse small intestine sections. It is not possible to resolve the lacteal in this image. (**C, D**) Identification of the lacteal in the villi by immunostaining with the anti-Lyve1 antibody (Green: Lyve1, Blue: Hoechst).

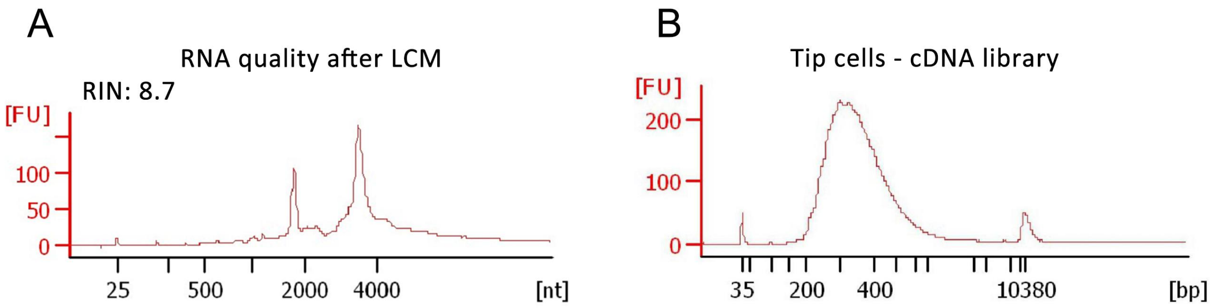

Figure S8: RNA and cDNA library quality check following the immuno-LCM steps during the collection of the mouse lacteal tip cells. (**A**) The RNA is well-preserved during this process. (**B**) The quality of the cDNA library generated from this RNA data is also high, as evidenced by the single distribution of sizes around 300 bp as expected.

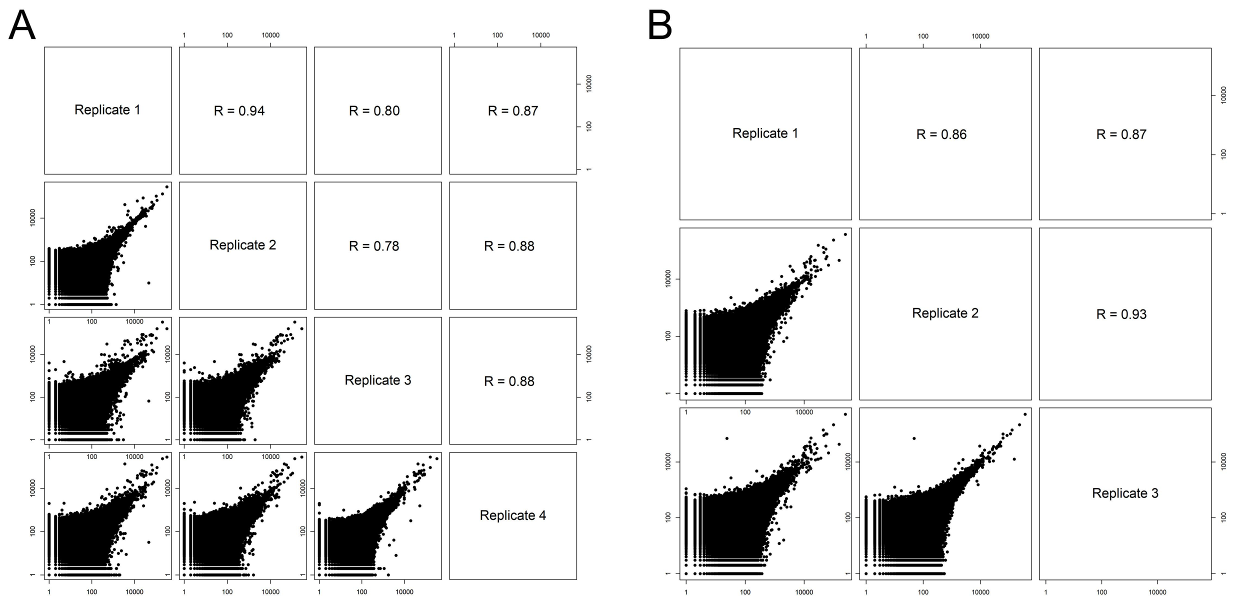

Figure S9: Reproducibility of the RNAseq data among lacteal tip or tube replicates samples. (**A, B**) Log-log scatter plots and Pearson correlation coefficients among the replicates of the lymphatic lacteal tube cell samples (**A**) and tip cell samples (**B**) from mouse small intestine frozen sections.

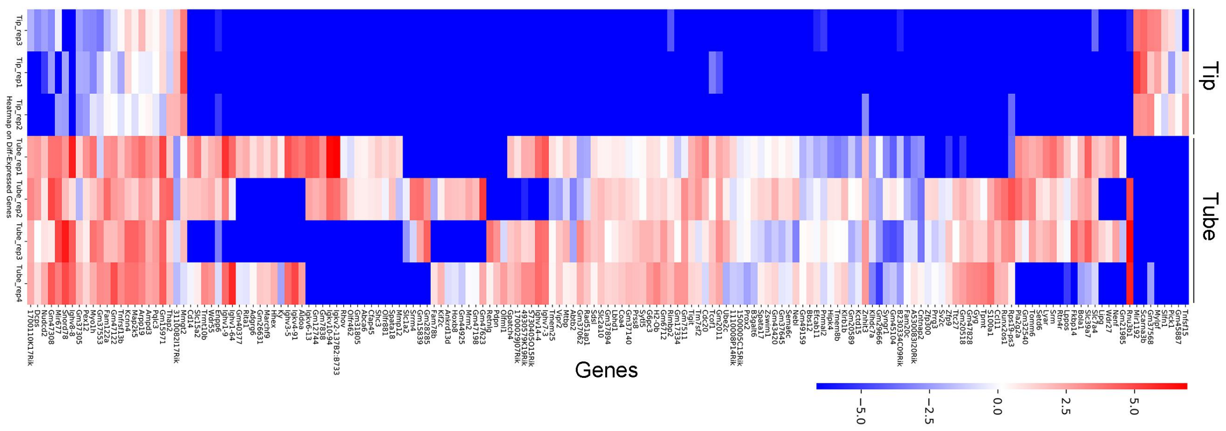

Figure S10: Differentially expressed genes between tip and tube samples. The 167 differentially expressed genes ($\log_2$(foldchange) > 2 or < -2, p_{adj} < 0.01) between the tip and the tube cells from the mouse small intestine lacteals.

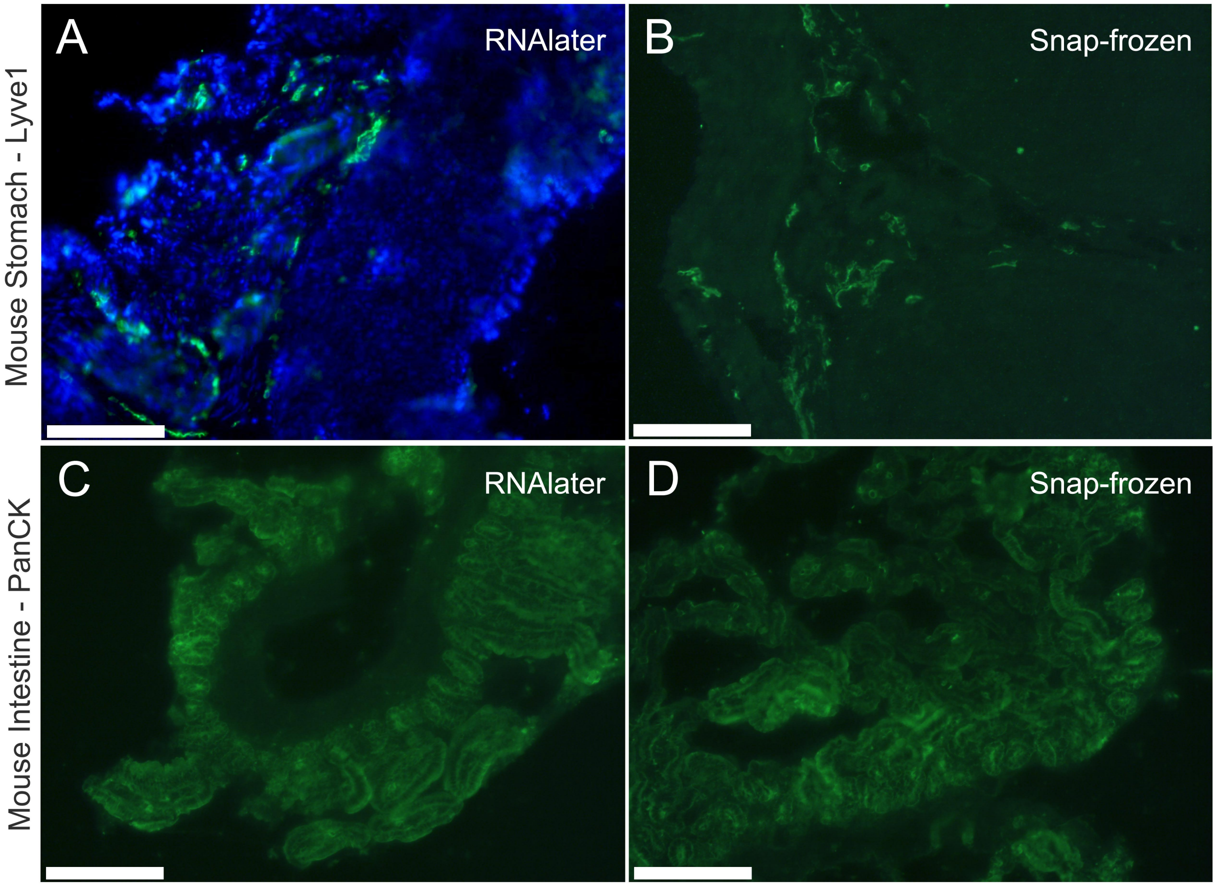

Figure S11: IF image quality following the RVC-based IF with RNAlater-preserved or snap-frozen tissue sections. (**A, B**) Lymphatic vessels labeled with anti-Lyve1 antibody from RNAlater-preserved (**A**) and snap-frozen (**B**) mouse stomach sections. (**C, D**) Epithelial cells labeled with anti-cytokeratin antibody (PanCK) with RNAlater-preserved (**C**) and snap-frozen (**D**) mouse small intestine sections. Scale bar: 200 µm.

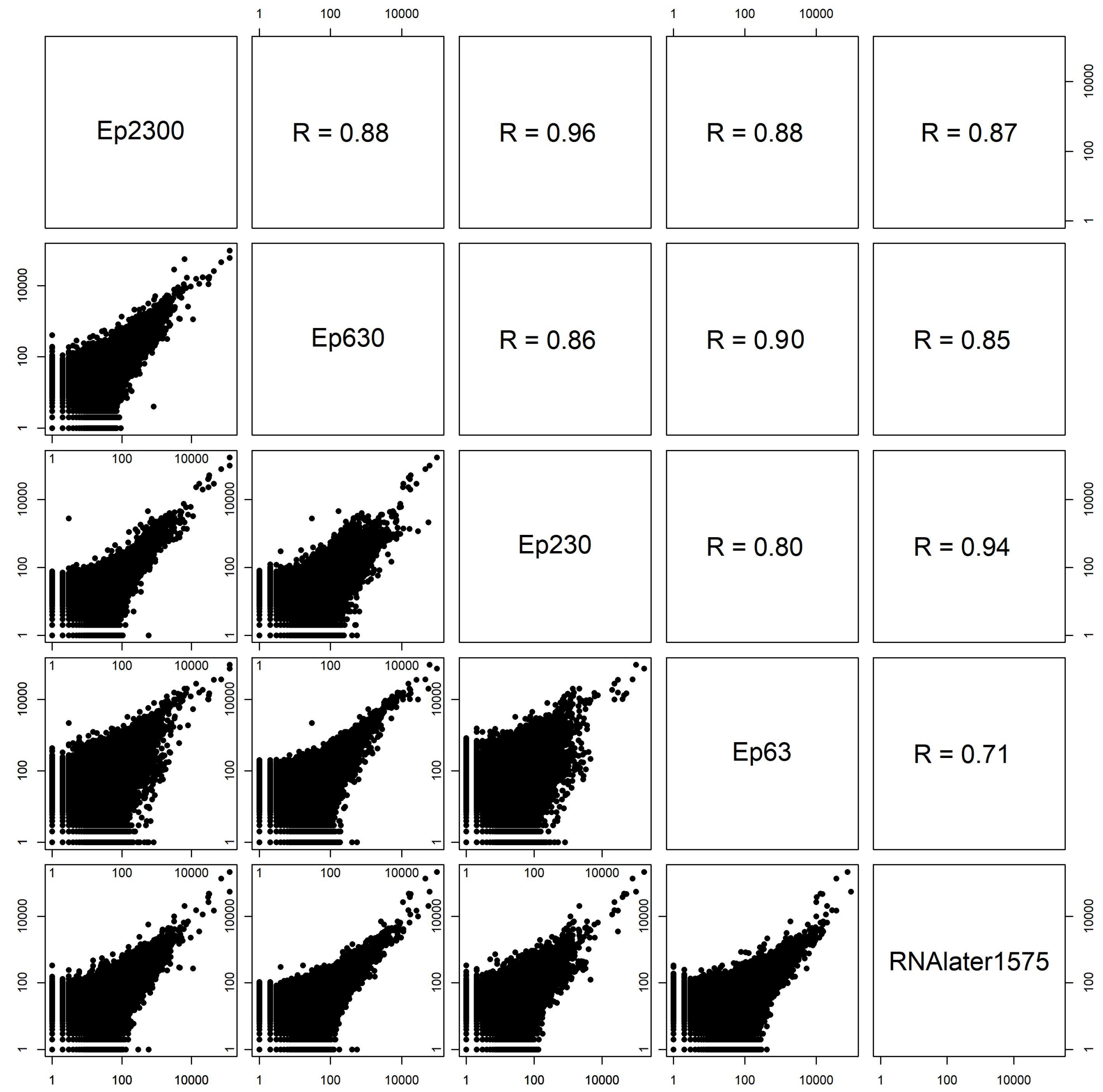

Figure S12: Reproducibility of the RNAseq data between RNAlater-preserved and snap-frozen preserved mouse intestine sections. Log-log scatter plots and Pearson correlation coefficients between the RNA data from the ~1575 Ep cells that were collected from the RNAlater-preserved sections and that obtained from the snap-frozen preserved sections. All these data were obtained from the crypt region in the mouse small intestine epithelial cells by immuno-LCM-RNAseq.

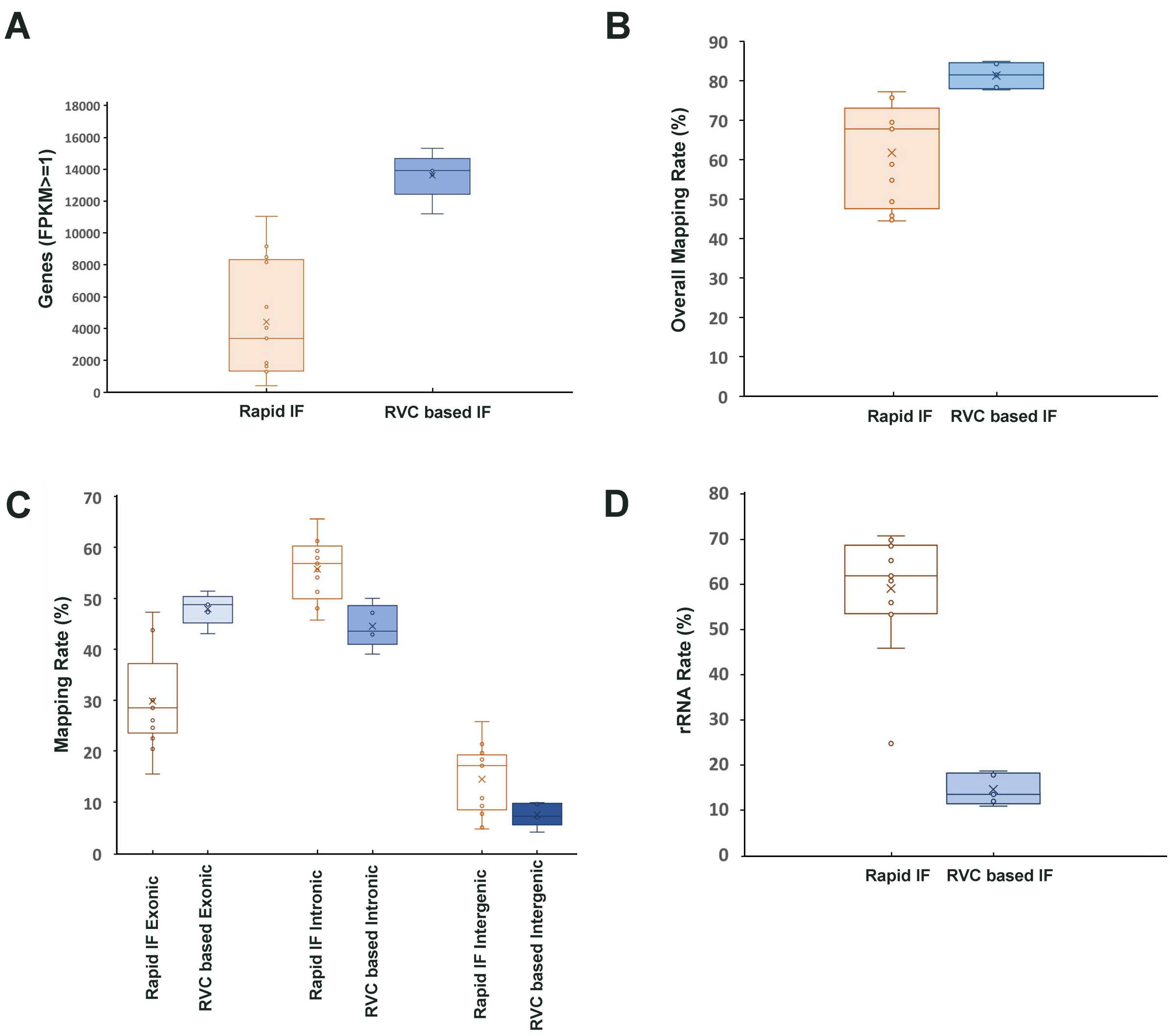

Figure S13: Comparison of the RNAseq data obtained with our method (small intestine epithelial cells) with that obtained recently using the Rapid protocol (Baccin et al. 2020)**.** Boxplots show the differences in (A) overall number of detected genes, (B) overall mapping rate, (C) exonic, intronic, and intergenic mapping rate, and (D) rRNA mapping rate between the two approaches. The analysis derived from the data in (Baccin et al. 2020) are from 13 different samples, 9 with a similar read depth as our samples and 4 with 50% greater read depth.

Table S1: Number of cells from PanCK-labelled epithelial cells (Ep) in the snap-frozen or RNAlater-preserved mouse intestine samples.

Samples	Total area (μm^2)	Effective areal fraction	Section thickness (μm)	Estimated cells number
Ep63	10800	0.68	12	63 cells
Ep230	40758	0.68	12	230 cells
Ep630	108000	0.68	12	630 cells
Ep2300	407580	0.68	12	2300 cells
RNAlater1575	270220	0.68	12	1575 cells

The average volume of intestine epithelial cells was calculated to be 1400 μm^3.

Cellular areal fraction refers to the fraction of the dissected region that is occupied by cells.

Estimated cells number = Total area * Effective areal fraction * Section thickness /1400 μm^3

Table S2: RNA sequencing information for all of our samples and 13 samples from a recently published Rapid Immunostaining-based LCM RNAseq study (Baccin et al. 2020).

Samples	Sequencing Clean Reads	rRNA Rate	Overall Mapped Rate	Exonic Rate	Intronic Rate	Intergenic Rate	Genes (FPKM ≥1)
Ep63	20.9M	12.00%	84.90%	48.77%	47.14%	4.08%	11201
Ep230	13.3M	17.80%	77.70%	51.39%	39.02%	9.59%	13657
Ep630	19.8M	10.90%	84.30%	43.07%	50.00%	6.94%	15315
Ep2300	15.6M	13.50%	78.30%	49.25%	43.54%	7.21%	13913
RNAlater1575	15.3M	18.70%	81.50%	47.25%	42.87%	9.88%	14019
H-RNAlater100	42.5M	23.00%	73.30%	45.94%	49.72%	4.34%	15819
Lacteal tip and tube samples							
Tip_replicate1	50.3M	22.42%	74.01%	50.25%	43.75%	6.00%	12389
Tip_replicate2	58.3M	17.77%	79.22%	45.77%	49.88%	4.35%	12345
Tip_replicate3	55.2M	16.91%	80.73%	47.34%	48.51%	4.15%	12598
Tube_replicate1	52.3M	19.61%	80.76%	55.54%	39.69%	4.77%	11771
Tube_replicate2	48.7M	15.43%	79.10%	48.18%	45.32%	6.49%	13069
Tube_replicate3	62.7M	15.88%	77.76%	42.23%	51.83%	5.94%	14509
Tube_replicate4	56.0M	14.40%	82.15%	41.95%	53.66%	4.39%	14223
Bone Marrow – Rapid immunofluorescence staining based LCM RNAseq samples							
S1	19.5M	68.82%	58.83%	22.99%	59.27%	10.73%	4043
S3	22.7M	55.98%	76.18%	43.74%	48.58%	7.68%	9167
S14	38.4M	53.68%	77.24%	43.75%	51.24%	5.01%	8487
S15	32.2M	45.90%	75.74%	47.26%	48.04%	4.70%	8164
S18	18.1M	68.56%	44.47%	24.57%	54.06%	21.37%	1298
S20	19.1M	69.87%	49.34%	22.48%	57.91%	19.61%	1348
S22	28.2M	61.91%	45.78%	28.49%	45.74%	25.77%	417
S28	21.0M	70.79%	68.05%	28.92%	61.85%	9.22%	3379
S29	27.5M	24.79%	70.41%	15.52%	65.56%	18.92%	11038
S42	21.2M	68.62%	67.79%	30.61%	58.44%	10.95%	1849
S49	19.2M	53.35%	69.46%	20.41%	61.25%	18.35%	5362
S75	22.7M	65.34%	44.70%	26.04%	56.81%	17.15%	1636
S77	20.5M	60.83%	54.79%	26.03%	55.51%	18.46%	1318

Table S3: Number of cells of Lyve1 labelled lacteal tip and tube samples from snap-frozen preserved mouse intestine and Podoplanin-labelled lymphatic vessel cells from the RNAlater-preserved clinical human jejunum sample.

Samples	LCM captured unit	Cells per unit	Section thickness (μm)	Species	Estimated cells number
**Tip_replicate1**	101 tips	1-2 cells	12	Mouse	~150 cells
**Tip_replicate2**	103 tips	1-2 cells	12	Mouse	~150 cells
**Tip_replicate3**	100 tips	1-2 cells	12	Mouse	~150 cells
**Tube_replicate1**	28 tubes	5-7 cells	12	Mouse	~170 cells
**Tube_replicate2**	27 tubes	5-7 cells	12	Mouse	~170 cells
**Tube_replicate3**	29 tubes	5-7 cells	12	Mouse	~170 cells
**Tube_replicate4**	28 tubes	5-7 cells	12	Mouse	~170 cells
**H-RNAlater100**	11 LCM unit	8-10 cells	12	Human	~ 100 cells

Table S4: Detected isoforms of Arpc1a in the tip and tube samples.

	**Transcript ID**	**Length (bp)**	**Annotation**	**Expression (FPKM)**						
				**Tip_ rep1**	**Tip_ rep2**	**Tip_ rep3**	**Tube_ rep1**	**Tube_ rep2**	**Tube_ rep3**	**Tube_ rep4**
**Arpc1a-201**	**ENSMUST00000031625**	**1632**	**Protein coding**	**0**	**0**	**0**	**33.43**	**14.63**	**19.08**	**29.10**
Arpc1a-202	ENSMUST00000124379	588	Protein coding	0	0	0	0	0	0	0
Arpc1a-203	ENSMUST00000127694	1685	Nonsense mediated decay	7.48	11.28	16.62	0.38	0.73	0	1.82
Arpc1a-204	ENSMUST00000134835	326	Processed transcript	0	0	0	0	0	0	0
Arpc1a-205	ENSMUST00000142276	926	Retained intron	15.15	3.83	10.19	0.52	6.50	6.80	2.13
Arpc1a-206	ENSMUST00000147564	425	Retained intron	0	0	1.26	0	3.27	0	1.96
